# Overcoming Incomplete Digestion of Ti-Doped LiFePO_4_ Cathode Materials: Microwave-Assisted Sample Preparation and Single-Laboratory Validated ICP-OES Determination of Li, Fe, P, and Ti

**DOI:** 10.3390/molecules31213915

**Published:** 2026-10-30

**Authors:** Myung-Gyu Lee, Tae-Young Kim, Sam-Hang Yi, Joo-Seok Park, Young-Jun Yoon, Kyoung-Seok Moon

**Affiliations:** 1 School of Materials Science & Engineering, Gyeongsang National University, Jinju 52828, Republic of Korea; lmk7251@kicet.re.kr (M.-G.L.); xboom0425@kicet.re.kr (S.-H.Y.); 2 Industry Support Division, Korea Institute of Ceramic Engineering & Technology, Jinju 52851, Republic of Korea; tykim@kicet.re.kr (T.-Y.K.); pjuju@kicet.re.kr (J.-S.P.); 3 Advanced Materials Technology Division, Korea Testing & Research Institute, Gwacheon 13810, Republic of Korea; yjyun@ktr.or.kr

**Keywords:** LiFePO_4_, cathode material, titanium doping, microwave digestion, ICP-OES, method validation, measurement uncertainty

## Abstract

Reliable compositional quality control of Ti-doped LiFePO_4_ (LFP) requires quantitative release of the low-level Ti dopant from a carbon-coated phosphate matrix. We evaluated aqua regia hot-plate digestion and developed a closed-vessel microwave digestion–inductively coupled plasma optical emission spectrometry (ICP-OES) method for Li, Fe, P, and Ti. Aqua regia left a carbon-rich residue containing Ti; mass balance on seven digestions showed that 5.86 ± 0.37% of total Ti was retained, compared with 0.39 ± 0.12%, 0.53 ± 0.11%, and 0.81 ± 0.13% for Li, Fe, and P. The mean Ti recovery across the 14 aqua regia digestions was 92.8% of the assigned value, compared with 100.0% for the microwave procedure. Digestion with 6 mL HNO_3_ and 2 mL HCl at 240 °C gave quantitative Ti release: between 220 and 240 °C the Ti result rose by 14.0% to a plateau, while Li, Fe, and P changed by less than 2%. Calibration R^2^ values exceeded 0.999. Recoveries were 98.6–99.8%, repeatability RSDs 0.49–1.51%, and relative expanded uncertainties 0.64–2.20%; the lower calibration limit for Ti was 250 mg kg^−1^. Method means agreed with values assigned by 14–16 laboratories (compatibility indices ≤ 0.49). Under routine conditions, the method would support Ti specifications of ±13.4% relative or wider.

## 1. Introduction

Lithium iron phosphate (LiFePO_4_, LFP) is an olivine-structured cathode material in which strong covalent P–O bonds within the PO_4_ polyanion framework contribute to structural and thermal stability during electrochemical cycling [1,2]. Its practical power capability, however, is limited by intrinsically low electronic and Li-ion conductivities. Particle downsizing, conductive carbon coating, and elemental doping are therefore widely used to improve rate capability and facilitate practical materials processing [3,4,5,6,7]. Titanium is among the dopants investigated for modifying the structural and interfacial properties of LFP; its concentration is therefore a relevant compositional and process-control parameter in Ti-doped LFP products [7].

Reliable compositional quality control of LFP requires accurate determination of the major constituents Li, Fe, and P together with the substantially lower Ti dopant concentration. ICP-OES is well suited to this task because it combines multi-element capability with a broad working range. In solution-based elemental analysis, however, overall method performance depends not only on the spectrometer but also on quantitative and reproducible sample decomposition. Carbon-coated LFP may not be quantitatively decomposed by conventional open-vessel acid treatment, and even a visually minor residue may selectively retain a low-level dopant. Published work has described ICP-OES determination of Li, Fe, and P in LFP after strong-acid digestion [8], but to our knowledge, no previous study has jointly quantified dopant retention in the digestion residue and integrated residue mass balance, matrix-specific wavelength selection, precision, and measurement uncertainty for Ti-doped industrial LFP. A certified reference material has recently been developed for the elemental composition of Li–Ni–Mn–Co oxide cathode material [9]; however, no publicly available matrix-matched certified reference material for Ti-doped LFP was identified at the time of this study.

A Korean standard for chemical analysis of lithium nickel manganese cobalt oxide (NMC), KS L 1629-1, specifies aqua regia-based hot-plate digestion [10]. Because NMC and carbon-coated LFP differ in crystal chemistry and surface treatment, direct application of this procedure to LFP requires matrix-specific verification. Method validation is required when a procedure is developed, modified, or applied outside its established scope [11,12,13,14,15]. In addition to trueness and precision, an LFP-specific method should evaluate matrix-induced spectral bias, establish digestion conditions that provide reproducible analyte release, distinguish post-digestion spike recovery from evidence of solid-sample digestion completeness, and characterize method robustness and measurement uncertainty.

Standards specific to LFP chemical analysis do exist. The YS/T 1028 series [16] specifies total iron by titanium trichloride reduction and dichromate titration, lithium by flame photometry, phosphorus by quinoline phosphomolybdate gravimetry, carbon by high-frequency combustion, and eleven trace impurities by ICP-OES; the corresponding product standard [17] likewise specifies no titanium content. The major constituents are therefore determined by classical methods, ICP-OES is applied only to impurities, and titanium is not among the elements covered. In Korea, KS L 1629-1 [10] was issued in 2023 as the first national standard for the chemical analysis of lithium secondary battery cathode materials; it covers NMC and determines seventeen elements, titanium included, by ICP-OES after acid decomposition, with further parts anticipated for other cathode chemistries. Recent instrumental work on battery-material quality control has likewise been benchmarked against these national regulations rather than against an LFP-specific ICP-OES procedure [18]. To our knowledge, no current standard provides a single ICP-OES procedure covering both the major constituents and a titanium dopant in carbon-coated LFP. In practice, laboratories adapt procedures written for a different matrix, which is why the aqua regia hot-plate digestion prescribed in KS L 1629-1 was taken as the starting point in this work, and why its applicability to a carbon-coated LFP matrix required verification.

The objectives of this work were therefore to: (i) determine whether aqua regia hot-plate digestion quantitatively dissolves the investigated commercial Ti-doped LFP material; (ii) establish a closed-vessel microwave digestion procedure compatible with ICP-OES determination of Li, Fe, P, and Ti; (iii) evaluate selectivity, working range, linearity, sensitivity, trueness, repeatability, intermediate precision, ruggedness, and measurement uncertainty; and (iv) compare the resulting method means with property values assigned to the same material through a multi-laboratory characterization exercise as an external check of method performance. The study focuses on a single commercial lot of carbon-coated Ti-doped LFP, thereby evaluating the complete analytical procedure in an actual product matrix rather than in synthetic solutions alone.

## 2. Results and Discussion

### 2.1. Failure of Conventional Aqua Regia Digestion

Application of the aqua regia hot-plate procedure to the Ti-doped LFP produced a black residue on the filter paper in every replicate. FE-SEM revealed a compact, heterogeneous solid. Across the individual EDS outputs, carbon was the dominant constituent (68–76 wt%); the mass-fraction results averaged 73.03 ± 2.65 wt% C, 3.52 ± 0.74 wt% P, 1.66 ± 0.30 wt% Fe, and 0.30 ± 0.07 wt% Ti. Ti was detected at all six locations, and the Fe/P atomic ratio was only 0.22–0.34, confirming that phosphate-containing, Ti-bearing material remained after digestion. Because EDS is semi-quantitative and does not detect Li, these results were used to establish elemental retention rather than absolute phase composition.

**Figure 1:**
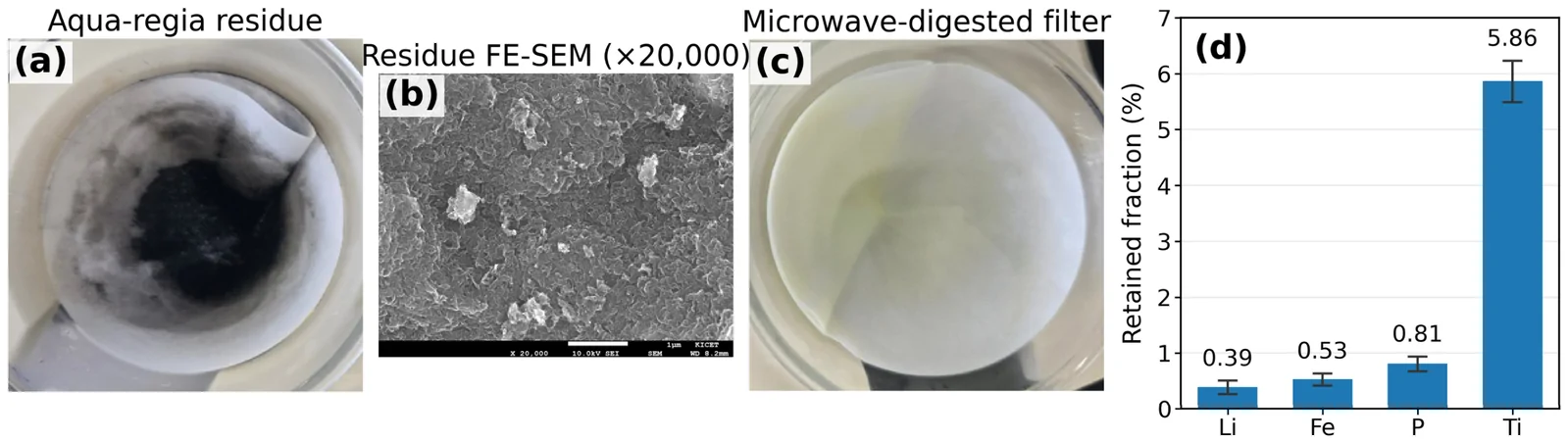
Evidence for selective analyte retention during aqua regia digestion of Ti-doped LFP: (**a**) black residue after open-vessel digestion; (**b**) FE-SEM image of the recovered residue; (**c**) filter after closed-vessel microwave digestion; and (**d**) fraction of each analyte retained in the aqua regia residue, determined by gravimetric and elemental mass balance on seven independent digestions (error bars: one standard deviation).

A quantitative mass balance was therefore performed on seven independent digestions. Each filter was dried and weighed before the retained solids were ashed and dissolved with HF and HCl, so that residue mass and residue composition were obtained from the same test portion. The recovered residue amounted to 2.050 ± 0.179 wt% of the test portion. The proportions of total analyte retained were 0.39 ± 0.12% for Li, 0.53 ± 0.11% for Fe, 0.81 ± 0.13% for P, and 5.86 ± 0.37% for Ti (Figure 1). Relative to Fe, Ti was enriched by a factor of 11.0. The conventional procedure therefore introduces a digestion-related negative bias for Ti that exceeds the ±5% acceptance interval used for the dopant, while its effect on the three major elements remains an order of magnitude smaller. These data demonstrate why visual confirmation of bulk dissolution alone is insufficient when a low-level dopant is the measurand.

Because filtrate and residue were measured on the same test portion, the mass balance can be verified directly. Summing the two fractions recovered 100.6% of the assigned Li, 98.1% of Fe, 99.9% of P, and 98.0% of Ti, so the retention values above are consistent with closure of the balance rather than inferred from the residue alone. The shortfalls for Fe (1.9%) and Ti (2.0%) are of similar size, so the small deficit is not specific to Ti; its cause was not identified. The retained fractions are therefore reported as measured, and possible losses during the residue work-up are noted as a limitation (Section 3.2).

Direct analysis of the aqua regia filtrates enabled comparison with the microwave digestion results. Expressed against the interlaboratory assigned values, aqua regia returned 100.6% for Li, 97.9% for Fe, 99.6% for P, and 92.8% for Ti, whereas the closed-vessel microwave procedure operated at 240 °C returned 100.4%, 99.9%, 100.0%, and 100.0% for the same elements. The −7.2% difference observed for Ti is thus a measured quantity; for the 5C set, whose residues were analyzed, the filtrates returned 92.1% of the assigned Ti value and the residue a further 5.86% (Table 1).

The EDS composition of the residue and the gravimetric mass balance describe the same material and should be reconciled. The measured residue mass removes the arithmetic difficulty that arises when the residue mass is instead back-calculated from the EDS mass fractions. With a measured residue of 2.05 wt% of the test portion, and taking the independently assigned total carbon content of 1.4846% as an upper bound, carbon can account for at most 72.4 wt% of the residue, which is compatible with the 73.03 ± 2.65 wt% returned by EDS. Beyond carbon, however, applying the localized EDS compositions to the bulk residue mass did not quantitatively reproduce the ICP-OES mass balance: on a test-portion basis the EDS compositions correspond to 0.034% Fe, 0.072% P and 0.0062% Ti, against 0.177%, 0.159% and 0.0084% measured after dissolution of the residue. This difference is attributable to the localized, substrate-sensitive and normalized nature of EDS. Loss on ignition of the residue at 500 °C, determined on the glass-fibre set, corresponded to 1.84 ± 0.11% of the test portion; this value exceeds the assigned total carbon content and therefore also includes adsorbed water and the decomposition of residual nitrate, and is reported as loss on ignition rather than equated with carbon content. EDS is semi-quantitative, samples only a small interaction volume, is normalized to 100% and does not detect Li; accordingly, the gravimetric ICP-OES mass balance carries the quantitative weight in this work, whereas EDS is used only to confirm the widespread presence of Ti, Fe and P in the residue.

Two filter media of different nominal retention were used in parallel. Residue mass fractions were 2.050 ± 0.179 wt% on quantitative cellulose filters (No. 5C) and 2.221 ± 0.152 wt% on glass-fibre filters (GF/F) (*p* = 0.08), and filtrate concentrations of Li, Fe, and P did not differ between the two sets; a marginal difference was observed for Ti (1.5% relative, *p* = 0.04), which is about five times smaller than the 7.2% difference between digestion procedures. The two filter media therefore produced closely similar results for Li, Fe, and P, whereas a small but statistically significant difference was observed for Ti. Loss of fine residue through the filter is consequently unlikely to account for the retention reported here, although it cannot be excluded at the level of a few per cent.

### 2.2. Closed-Vessel Microwave Digestion

Closed-vessel digestion with a nitric-acid-rich mixture was adopted to oxidize the carbon coating and decompose the phosphate matrix under elevated temperature and pressure. The acid charge was 6 mL HNO_3_ and 2 mL HCl. For Li, Fe, and P, approximately 0.1 g of sample was digested and diluted to 1000 mL; for Ti, 0.20 ± 0.01 g was digested and diluted to 100 mL to improve sensitivity. A 0.3 g Ti test portion occasionally yielded residual material at the same acid volume, whereas the 0.2 g test portion produced stable, residue-free digests in all trials. The element-specific test portions and final volumes positioned the expected sample concentrations near the middle of their respective calibration ranges.

The final microwave program consisted of a 30 min ramp to 240 °C, a 60 min hold, and 60 min cooling. The digests were clear and pale yellow, and no visible residue remained after filtration through No. 5C quantitative cellulose filter paper, the medium also used for the 5C set of the aqua regia test. However, the approximately 0.29 mg of Ti present in a 0.2 g test portion would not be visually discernible even if fully retained. The absence of visible solids was therefore treated as evidence of bulk decomposition, whereas quantitative release of Ti was evaluated jointly using the residue mass balance and the independent interlaboratory comparison described in Section 2.9.

### 2.3. Spectral Selectivity and Wavelength Selection

Selectivity was evaluated by holding each analyte at 10 mg L^−1^ while increasing the concentrations of the principal coexisting elements. For Li, Fe, and P, the matrix elements were varied from 0 to 100 mg L^−1^. For Ti, Li, Fe, and P were varied simultaneously from 0 to 1000 mg L^−1^ each, so that the total coexisting-element load at the highest level was approximately 3000 mg L^−1^, about 2.6 times that of the real Ti sample solution (approximately 1140 mg L^−1^). Selectivity was therefore assessed against the combined matrix rather than against each element in isolation. Four candidate wavelengths were investigated for each element.

**Figure 2:**
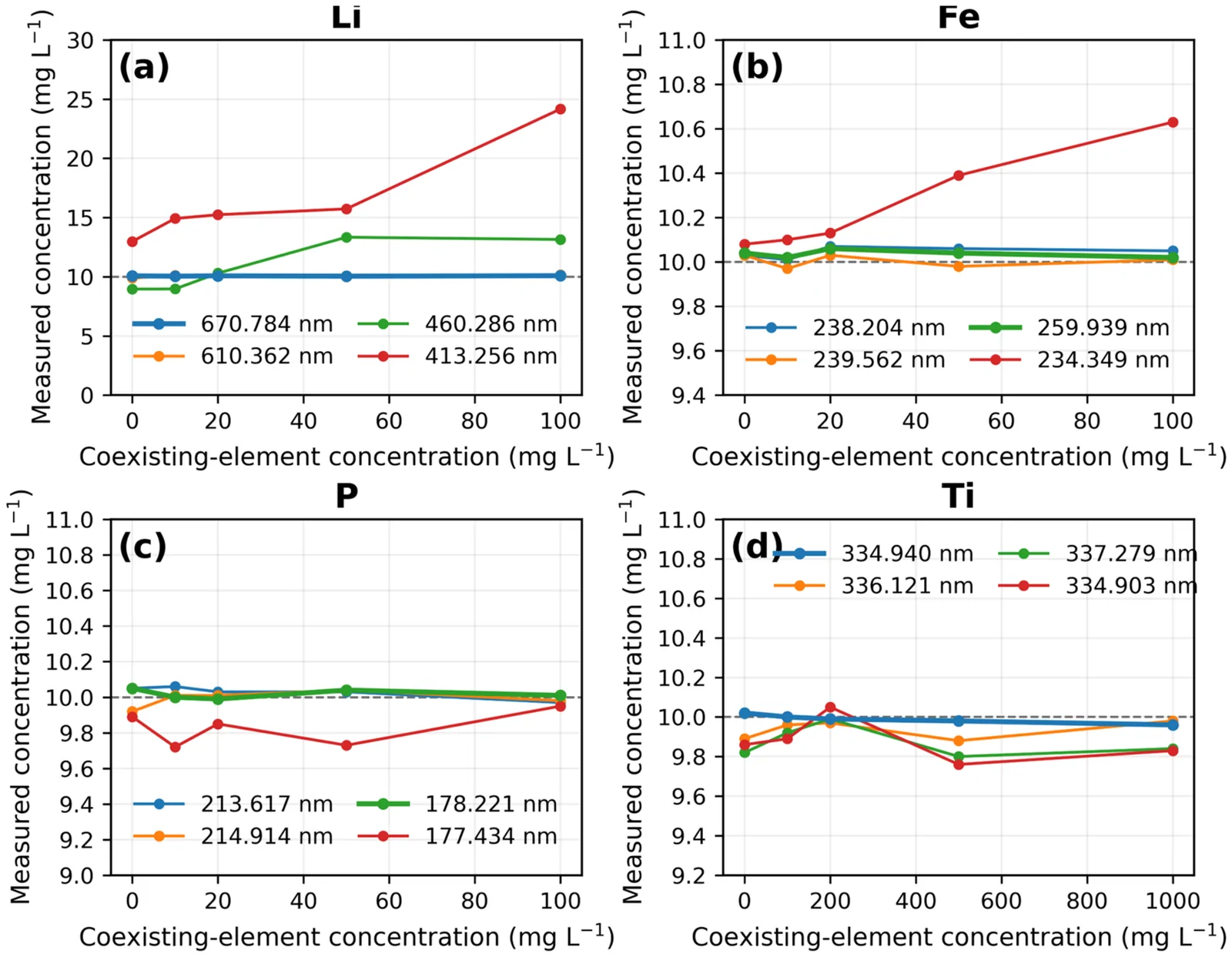
Selectivity assessment for (**a**) Li, (**b**) Fe, (**c**) P, and (**d**) Ti. The analyte concentration was 10 mg L^−1^. The thicker curve in each panel indicates the selected analytical wavelength.

The selected lines were Li 670.784 nm, Fe 259.939 nm, P 178.221 nm, and Ti 334.940 nm (Figure 2). Across the evaluated matrix ranges, their recovery intervals were 100.46–100.89%, 100.20–100.60%, 99.90–100.47%, and 99.56–100.22%, respectively. By contrast, Li 460.286 and 413.256 nm exhibited severe positive matrix-dependent deviations, Fe 234.349 nm increased to 106.32%, and P 177.434 nm showed a persistent negative deviation. For Ti 334.940 nm, the regression trend was statistically detectable (*p* = 0.007), but the predicted bias at the maximum matrix concentration was only −0.54%, which was analytically negligible relative to the ±5% acceptance interval. Wavelength selection based on both recovery and the confidence interval of matrix-dependent bias (Table S7) avoided relying on sensitivity alone.

The matrix load differs between the two dilution schemes, and each was covered separately. The Li/Fe/P sample solution (0.1 g in 1000 mL) contains about 57 mg L^−1^ of matrix elements in total, within the 0–100 mg L^−1^ per-element range tested for those analytes, whereas the Ti sample solution (0.2 g in 100 mL) contains about 1140 mg L^−1^, within the combined load of approximately 3000 mg L^−1^ tested for Ti. The twenty-fold higher matrix load of the Ti solution is therefore bracketed by the selectivity data, over which the magnitude of the predicted negative bias for Ti at 334.940 nm did not exceed 0.54% (Tables S6 and S7). Matrix effects at the actual Ti sample load were also captured by the post-digestion spike recovery (Section 2.5), which was performed in a matrix reproducing the Li, Fe, and P concentrations of a real Ti sample solution, and residual transport and plasma-loading effects were compensated by the Y internal standard.

### 2.4. Linearity and Sensitivity

Calibration curves were fitted over the measured calibration standards. The blank-corrected origin was not treated as a calibration point, since its value is fixed at zero by the blank correction and it carries no independent information. Seven non-zero calibration levels were used for Li, Fe, and P. For Ti, the 0.1 mg L^−1^ level was excluded because it lay +29.8% above the extrapolated calibration (individual calibrations +22.5% to +34.3%). The working ranges used for quantification were 1–7 mg L^−1^ for Li, 5–60 mg L^−1^ for Fe, 5–35 mg L^−1^ for P, and 0.5–5 mg L^−1^ for Ti. The expected sample concentrations occupied 49–55% of these ranges. All intensities were corrected using Y at 5 mg L^−1^ as an internal standard and blank-subtracted.

**Figure 3:**
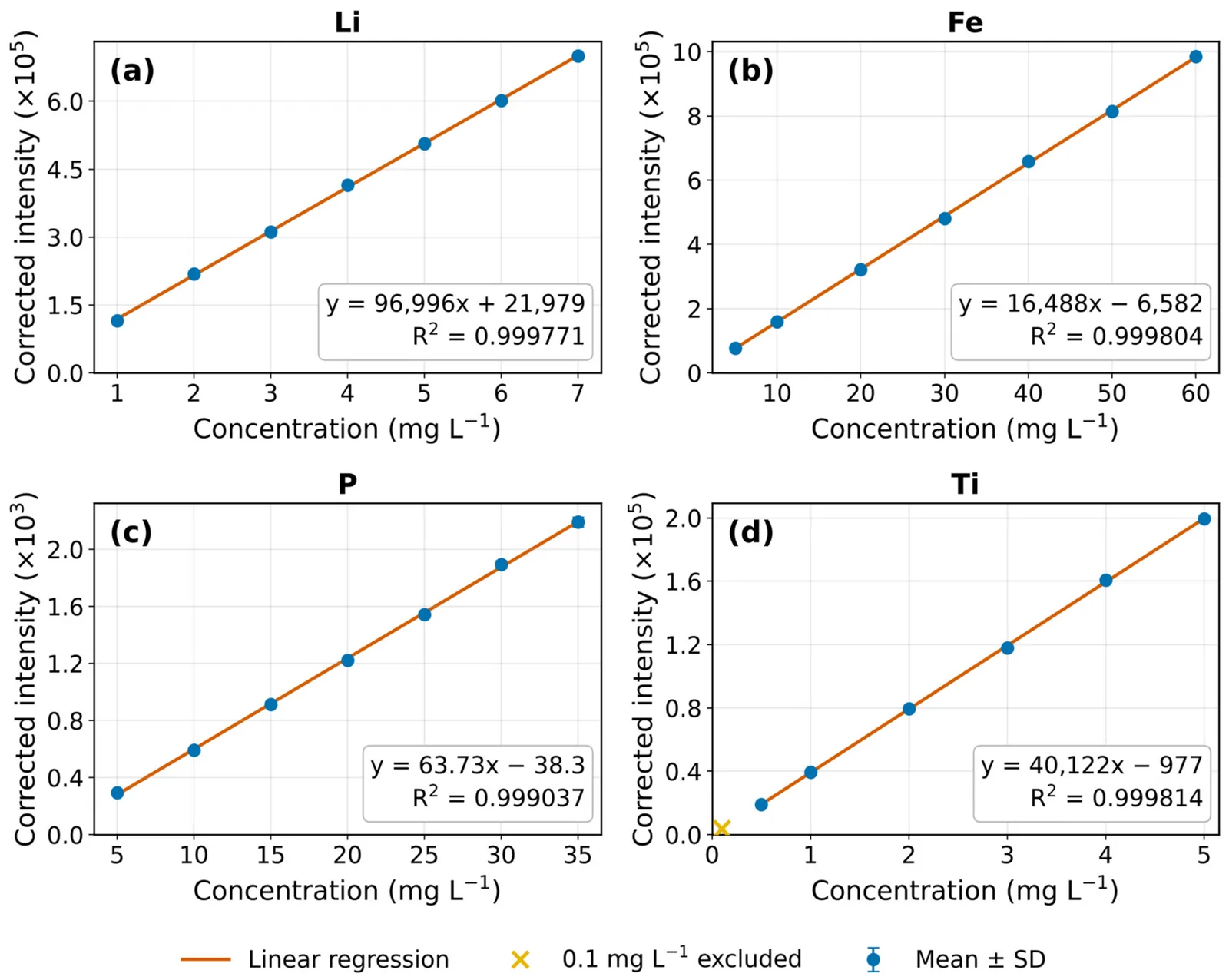
Calibration curves for (**a**) Li, (**b**) Fe, (**c**) P, and (**d**) Ti using mean internal-standard-corrected intensities. The blank-corrected origin is not treated as a calibration point. The 0.1 mg L^−1^ Ti level is shown but was excluded from the final regression because it lay +29.8% above the extrapolated calibration.

The coefficients of determination were 0.999771, 0.999804, 0.999037, and 0.999814 for Li, Fe, P, and Ti, respectively (Figure 3, Table 2). More importantly, residuals near the sample concentrations were +1.1%, −1.6%, −1.0%, and −1.3%, within the concentration-dependent acceptance limits. Triplicate independently generated calibration slopes had RSDs of 0.23% for Li, 0.08% for Fe, 0.62% for P, and 0.13% for Ti, confirming stable sensitivity. The fitted intercepts are appreciable relative to the lowest standard of each range (+19.0% for Li, −8.5% for Fe, −13.0% for P, and −5.1% for Ti), indicating that the linear model is not exact at the bottom of the working ranges. Reported results are unaffected because the sample concentrations lie near the middle of their ranges, but the validated range is taken to begin at the lowest calibration standard, and results below it are not reported.

### 2.5. Trueness and Precision

Because a certified reference material for Ti-doped LFP was not available at the method-development stage, trueness for Li, Fe, and P was assessed using a laboratory-prepared material based on high-purity Li_2_O, Fe_2_O_3_, and P_2_O_5_. Seven independent preparations gave recoveries of 99.8% for Li, 99.3% for Fe, and 99.0% for P. Ti was evaluated by adding standard solution to the digest of the same laboratory-prepared material at sample-basis levels of 0.05%, 0.15%, and 0.25%. Because that digest was prepared from a 0.2 g test portion diluted to 100 mL, the spiked solutions contained Li, Fe, and P together at approximately 88, 708, and 393 mg L^−1^, which reproduces the matrix load of a real Ti sample solution (87, 661, and 392 mg L^−1^), while Ti was present at 1.0–5.0 mg L^−1^, spanning the working concentration of the sample solution (2.85 mg L^−1^). The matrix contained no titanium, so no correction for a native contribution was required. Mean recoveries were 98.6%, 99.6%, and 99.1%, respectively; all 21 individual values were 97.6–100.9%, and no significant concentration dependence was observed (*p* = 0.27).

These trueness experiments address different parts of the analytical process. The synthetic Li–Fe–P material reproduces elemental composition but not the LFP crystal phase or carbon coating. Likewise, Ti was added after digestion and therefore tests dilution, internal-standard correction, calibration, and matrix effects after dissolution; it does not by itself demonstrate release of lattice- or coating-associated Ti. Digestion completeness was evaluated independently by residue mass balance and the interlaboratory method comparison. A solid control prepared by adding a Ti compound such as TiO_2_ to LFP was not used for the same reason: it would test the dissolution of the added compound, which is itself refractory, rather than the release of Ti incorporated in the doped, carbon-coated particles. Undoped LFP materials carry no assigned Ti value, and no Ti-doped LFP reference material was available, so the interlaboratory characterization of the same lot (Section 2.9) served as the matrix-matched external check. This separation prevents post-digestion spike recovery from being overinterpreted as extraction recovery for Ti bound in the solid.

**Figure 4:**
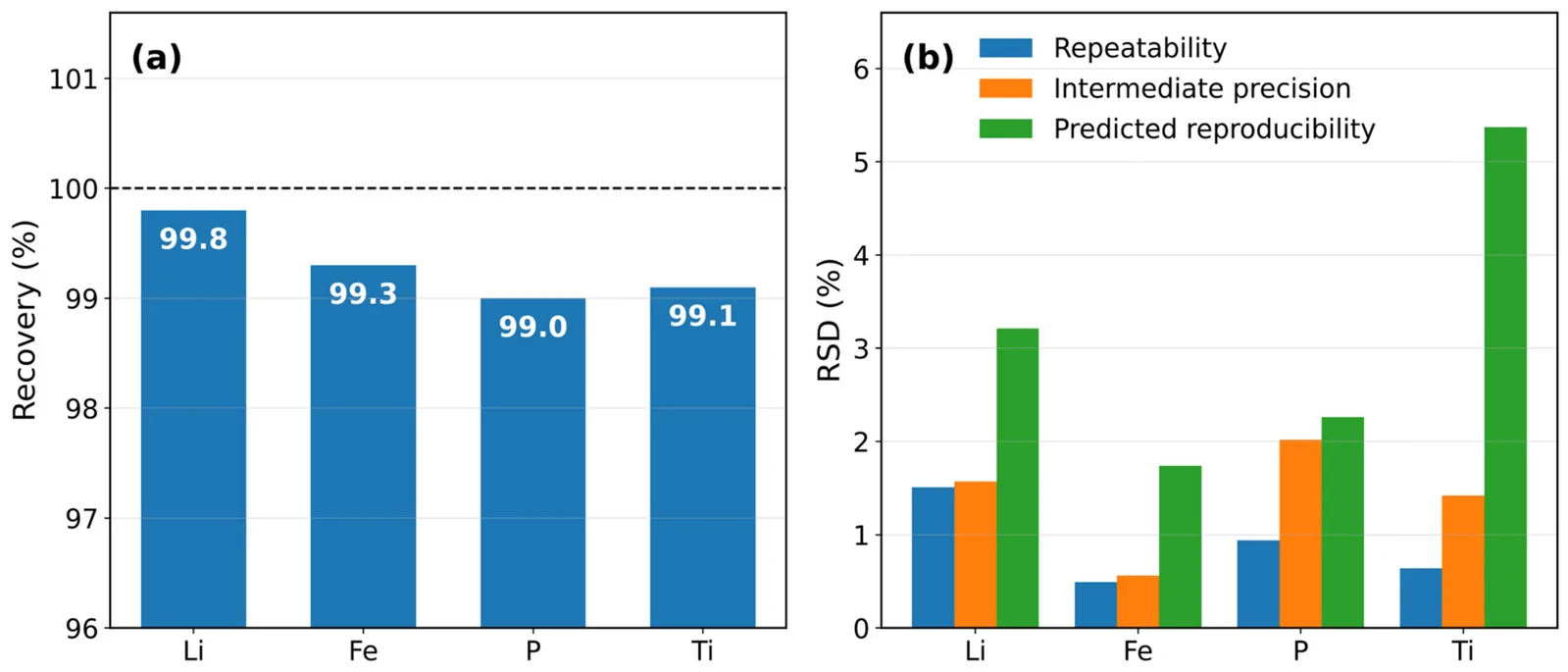
Validation performance: (**a**) mean recoveries for Li, Fe, P, and Ti (the Ti value is the mean across three spike levels); and (**b**) repeatability and intermediate-precision RSDs compared with Thompson-modified Horwitz predicted reproducibility.

Repeatability was evaluated through seven complete, independent weigh–digest–dilute–measure cycles. Means were 4.3505% Li, 33.062% Fe, 19.582% P, and 0.14073% Ti, with RSDs of 1.51%, 0.49%, 0.94%, and 0.64%, respectively. Intermediate precision involved three analysts, each completing seven independent determinations. Conventional one-way ANOVA yielded *p* = 0.144, 0.062, 0.141, and 0.880 for Li, Fe, P, and Ti. A Welch sensitivity analysis placed Fe just below and P just above the 0.05 threshold (*p* = 0.047 and 0.052, respectively). Nevertheless, the largest analyst-mean differences were only 0.60% relative for Fe and 1.88% for P, both within the ±2% practical bias interval. The combined intermediate-precision RSDs were 1.57%, 0.56%, 2.02%, and 1.42%, all below the corresponding Thompson-modified Horwitz predicted reproducibility values (Figure 4, Table 3).

### 2.6. Working Range and Lower Calibration Limit for Ti

The lower calibration limit for Ti in this method is the lowest calibration standard, 0.5 mg L^−1^, corresponding to 250 mg kg^−1^ for a 0.2 g test portion diluted to 100 mL; results are not reported below it. Method performance was characterized primarily at the sample concentration: the Ti content of the commercial sample, approximately 1400 mg kg^−1^, is about 5.6 times the lower calibration limit, so the working range is adequate for the intended compositional quality-control application. Detection capability, in the sense of the IUPAC nomenclature for detection and quantification capabilities [19], was not established below the calibrated range. The spiking experiment described in the Supplementary Information was measured against the routine 0.5–5.0 mg L^−1^ calibration, and the 0.1 mg L^−1^ Ti level had already been excluded from that calibration because of its residual; values derived from those measurements would therefore rest on extrapolation below the point at which the calibration model was shown to fail, and on solution concentrations quantized by the reporting resolution. They are reported only as an indication of sensitivity under routine conditions and are not used to define a limit of detection or quantification. Procedural blanks support this scope: the Ti reading of the procedural blank was typically 0.009 mg L^−1^, equivalent to 4.5 mg kg^−1^ on a solid basis, below 2% of the lower calibration limit and below 0.4% of the sample concentration.

Performance at the lower calibration limit itself was verified with a matrix-matched check solution containing 0.5 mg L^−1^ Ti, prepared from a stock solution independent of that used for the calibration standards and measured seven times against the routine calibration (Table S33). The mean found was 0.4996 mg L^−1^, a recovery of 99.9%, with an RSD of 1.32%, within acceptance criteria of 95–105% recovery and an RSD of no more than 10%. The uncertainty estimate combined the repeatability of the lower-limit check solution with the complete-method repeatability at the sample concentration and the mass, volume, and calibration-prediction terms of the uncertainty model in Section 2.8; the complete-method repeatability was included as a conservative allowance for variability associated with the digestion step. The resulting estimated relative expanded uncertainty (k = 2) for a single result was 8.7% at 250 mg kg^−1^, compared with 1.9% at the Ti content of the investigated material (Table S34). At 250 mg kg^−1^, calibration prediction accounted for 88% of the combined variance. Because the check was made on a solution rather than on a solid containing Ti at this level, this figure is a model-based estimate for the investigated matrix; on that basis, Ti contents down to 250 mg kg^−1^ are expected to be reportable with a single-day relative expanded uncertainty below 10%, which should be confirmed with a low-Ti solid material where such results are reported routinely. Where tighter control is required at low Ti contents, a larger test portion or a calibration range extended downward would need to be validated.

### 2.7. Ruggedness: Digestion Temperature as a Critical Control Factor

**Figure 5:**
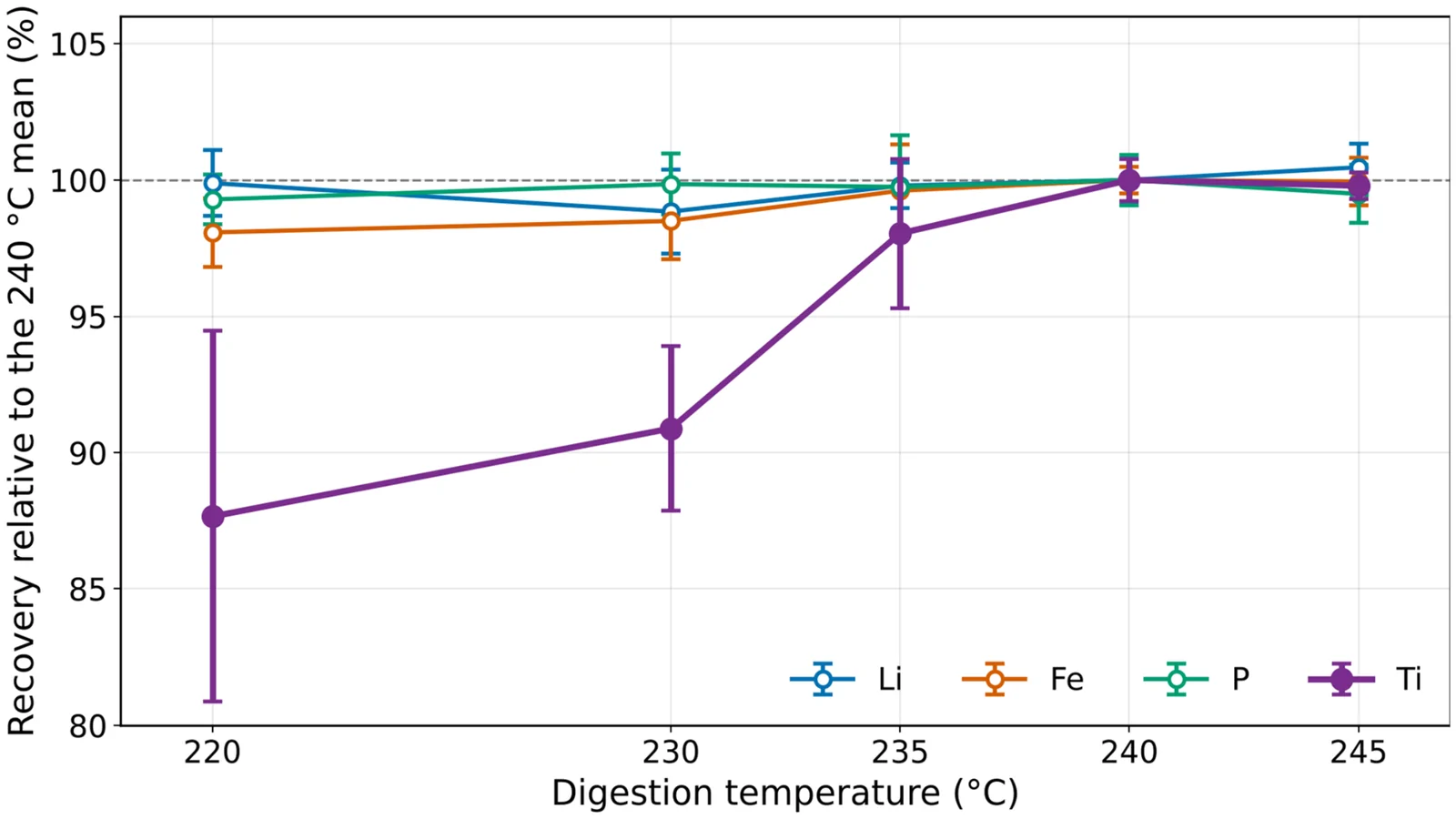
Effect of digestion temperature on analyte release (n = 7 per level, error bars: one standard deviation): recovery of Li, Fe, P, and Ti relative to the 240 °C mean. Photographs of the digests are given in the Supplementary Information.

Microwave temperature was varied at five levels between 220 and 245 °C, with seven independent digestions at each level, while the test portion, acid composition, ramp, and hold time were held constant. All digests were filtered under identical conditions irrespective of appearance, and Li, Fe, P, and Ti were determined in every filtrate. Titanium showed a clear dose–response (Figure 5). Relative to the 240 °C mean, recovery was 87.7% at 220 °C, 90.9% at 230 °C, 98.1% at 235 °C, 100.0% at 240 °C, and 99.8% at 245 °C, an increase of 14.0% between 220 and 240 °C. The three major elements changed by 0.1% (Li), 0.7% (P), and 1.9% (Fe) over the same interval, so the transition is specific to the dopant and not a general dissolution effect. Comparing adjacent temperature levels by Welch two-sample t-tests, the step occurs between 230 and 235 °C (*p* = 0.0005), whereas 235 and 240 °C do not differ significantly (*p* = 0.12) and neither do 240 and 245 °C (*p* = 0.52). The 240 °C set point therefore lies on the plateau of the response rather than on its shoulder and provides margin above the transition rather than defining it.

Precision followed the same pattern. The repeatability RSD for Ti was 7.78% at 220 °C, 3.30% at 230 °C, 2.77% at 235 °C, 0.75% at 240 °C, and 0.46% at 245 °C. Incomplete release of the dopant therefore degrades repeatability as well as trueness, so a method that relies on visual inspection alone may pass a precision check and still be biased. This experiment varied only temperature and therefore defines a set point for the investigated matrix and acid/test-portion ratio rather than a complete factorial robustness model for acid ratio, ramp time, hold time, or vessel design.

The temperature dependence is consistent with the chemistry of the coating rather than with thermal decomposition. In air, the carbon coating on the olivine particles combusts only at 520–540 °C [20], and the deposit formed at the surface of LFP grains is an amorphous graphitic carbon comparable to that obtained by high-temperature pyrolysis [21], which is correspondingly resistant to oxidation. At 220–245 °C, the coating is therefore removed not by combustion but by chemical oxidation with nitric acid, a reaction that requires temperatures above 200 °C to proceed extensively and that approaches its practical working range near 250 °C [22]. This is why a closed vessel is needed: it allows the acid to be held well above its atmospheric boiling point, and the oxidizing power of the acid rises steeply over this interval. Comparable behaviour has been reported for other carbonaceous matrices, where HF-free pressurized-cavity microwave digestion of recycled carbon fibre required temperatures of about 280 °C before refractory components dissolved [23].

While the coating remains intact, any Ti-bearing phase associated with it is shielded from the acid. Titanium oxides are practically insoluble in hydrochloric and nitric acid and dissolve appreciably only in hydrofluoric or hot concentrated sulfuric acid; in the HF-free digestion of a carbonaceous matrix, incomplete decomposition of rutile-type titania was in fact the factor that limited the reportable titanium content [23]. Titanium associated with the surface layer would then be released only after the carbon has been oxidized, whereas Li, Fe, and P were largely released under the tested conditions despite incomplete removal of the carbon-containing surface layer. The observed increase between 230 and 235 °C is consistent with sufficiently rapid nitric-acid oxidation of the carbon-containing surface layer and/or improved exposure of Ti-bearing regions under the specified digestion conditions, and the plateau above it with full exposure of the dopant. The Ti-bearing phase was not identified in this work, so this account is offered as a mechanism consistent with the observations rather than as a demonstrated pathway.

The 240 °C set of the temperature study gave a Ti mean of 0.14267%, compared with 0.14073% obtained in the repeatability study reported in Section 2.5. The two datasets are compatible (En = 0.91), and the temperature-study mean lies closer to the assigned value (−0.03% against −1.39%); both are retained as measured, and the repeatability figures quoted elsewhere in this work refer to the earlier dataset.

### 2.8. Measurement Uncertainty

The uncertainty model included test-portion mass, final volume, calibration prediction, and repeatability. Components common to replicate measurements (mass, volume, and calibration) were separated from the random repeatability component to avoid double counting. The uncertainty of the test-portion mass combines the repeatability of replicate weighings (8.4 × 10^−5^ g, n = 10), the calibration certificate of the check weight (1.5 × 10^−5^ g), the calibration certificate of the balance (1.0 × 10^−4^ g), and the balance resolution (5.8 × 10^−5^ g), giving 1.44 × 10^−4^ g for a 0.1 g test portion. The uncertainty of volume is not that of a single flask: for the 1000 mL sample dilution it combines the repeatability of the filling operation (0.045 mL, n = 10), the calibration certificate of the flask (±0.18 mL at k = 2, hence 0.090 mL), and a temperature term, while preparation of the calibration standards contributes a further 0.109 mL. Combined standard uncertainties were 0.0291% for Li, 0.1062% for Fe, 0.2156% for P, and 0.00105% for Ti; expanded uncertainties (k = 2) were 0.058%, 0.213%, 0.431%, and 0.0021%, corresponding to relative expanded uncertainties of 1.34%, 0.64%, 2.20%, and 1.49%, computed from the unrounded combined values (Table 4). The effective degrees of freedom were 10.7, 28.9, 24.7, and 26.5; k = 2 is therefore an approximation to the corresponding Student’s t values, retained for consistency with routine reporting practice.

**Figure 6:**
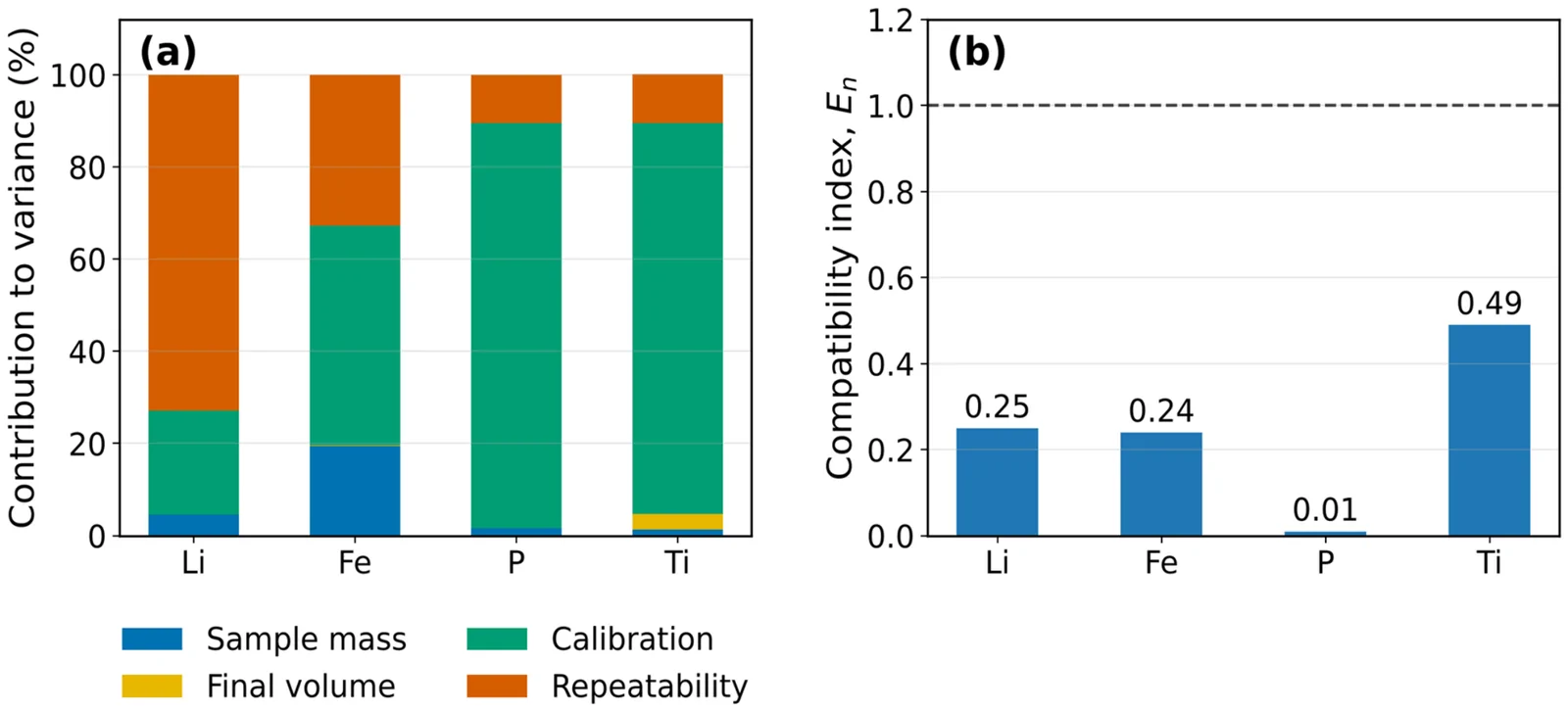
(**a**) Relative contribution of each component to the method-uncertainty variance; (**b**) agreement of the method mean with the interlaboratory assigned value, expressed as the compatibility index E_n_ = |D|/√(U_ref^2^ + U_method^2^). The dashed line indicates E_n_ = 1.

The component structure differed among analytes (Figure 6a). Repeatability accounted for 72.9% of the Li variance, whereas calibration dominated Fe (47.6%), P (87.9%), and Ti (84.8%). Two factors act together for P. The vacuum-UV line at 178.221 nm gives a response about two orders of magnitude lower than the other analytical lines, so counting statistics dominate its calibration prediction; in addition, axial viewing was adopted for P in order to recover that sensitivity, and axial viewing is more susceptible to matrix effects than radial viewing in a solution containing several hundred mg L^−1^ of Fe and P. Neither factor compromises trueness—P agreed with its assigned value to −0.04%, the closest of the four analytes—but both act on precision, and they are consistent with P showing the largest calibration contribution, the largest relative expanded uncertainty, and the only failure of the equal-variance checks in the intermediate-precision study. The Ti result reflects prediction uncertainty at a lower solution concentration. Because the read time is already at its automatic maximum for the weakest line, improvement is more likely to come from increasing the number of calibration levels and of readings per solution, from bracketing calibration or gravimetric dilution of the standards, or from matrix-matched calibration for P, than from tighter control of weighing or volumetric operations.

The budget above represents single-analyst, same-day conditions. For fitness-for-purpose under routine conditions, a second estimate was derived by combining the intermediate-precision variance with an uncorrected-bias term, following ISO 21748:2017 [24] and the Eurachem/CITAC guide [25]. The resulting relative expanded uncertainties (k = 2) were 3.17% for Li, 1.79% for Fe, 4.51% for P, and 3.36% for Ti. Reporting both figures is informative: the routine estimate for Ti covers the −1.39% difference from the assigned value with margin, which the single-day value of 1.49% barely does, and the routine estimate for P covers both the 1.88% largest analyst-mean difference and the 2.02% intermediate-precision RSD, neither of which is covered by the single-day budget.

### 2.9. Agreement with Interlaboratory Assigned Values

As an external check, method means were compared with property values assigned to the same material through a characterization exercise involving 14–16 laboratories. Participating laboratories used diverse decomposition and measurement approaches (Table S24); all applied closed-vessel pressure digestion, HF- or H_2_SO_4_-containing multi-acid treatment, or ashing followed by redigestion, and none relied on open-vessel aqua regia alone. No Grubbs outliers were identified, and all laboratory z-scores were within |z| < 2; with 14–16 laboratories, this criterion is weak evidence of consensus and is reported as a screening step. The assigned values weight laboratories equally regardless of the number of determinations contributed. Assigned values (expanded uncertainty, k = 2) were 4.2913 ± 0.227% Li, 33.301 ± 0.966% Fe, 19.590 ± 0.909% P, and 0.14272 ± 0.0034% Ti. These uncertainties combine the standard error of the laboratory-mean distribution (u_char) with between-bottle homogeneity (u_bb), long-term stability (u_lts) and short-term stability (u_sts) under ISO Guide 35:2017 [26], now superseded by ISO 33405:2024 [27]. Homogeneity was evaluated on ten bottles with three determinations each; long-term stability over six months at 20 °C by linear regression of the observed trend; and short-term stability at −10, 20 and 50 °C over two weeks, to cover transport and short-term storage. No short-term trend was resolved at any storage condition (*p* = 0.42–0.98), but a non-significant test is not itself a bound, so u_sts was evaluated as the standard error of the fitted slope multiplied by a two-week exposure and taken as the largest value across the three conditions: 0.019% for Li, 0.099% for Fe, 0.059% for P, 0.00041% for Ti and 0.0012% for C. It is carried as a component of the budget rather than set to zero and contributes 1.7–5.8% of the variance for the four analytes. The complete budget is given in the Supplementary Information. Long-term stability dominates, contributing 67–89% of the variance for the four analytes; this reflects the classical design of the stability study, in which successive time points were measured in different analytical runs, so that run-to-run variation is carried into the standard error of the fitted slope, and is not evidence of instability in the material.

Differences between the developed method and the assigned values were +1.38% relative for Li, −0.72% for Fe, −0.04% for P, and −1.39% for Ti. The three laboratories that used HF-containing multi-acid digestion, the most aggressive treatment represented in the comparison, returned a mean Ti value of 0.14278%, against 0.14270% for the other eleven laboratories, a relative difference of 0.05%. With n = 3 against n = 11 and s = 0.0030%, the smallest difference detectable at 80% power is about 0.0055%, or 3.8% relative; the comparison therefore excludes a loss on the 5.9% scale observed with aqua regia, which is the comparison that matters here, while residual Ti losses below about 4% would not be resolved by this test. Twelve of the sixteen participating laboratories, and ten of the fourteen reporting Ti, used closed-vessel microwave digestion, so part of this agreement is with laboratories that share the same technique class. Two participants are affiliated with the authors, as declared; recomputing the assigned values with those two laboratories removed changed them by +0.18% for Li, −0.09% for Fe, +0.20% for P, and −0.13% for Ti, in every case less than 6% of the corresponding assigned expanded uncertainty, and the compatibility indices became 0.22, 0.21, 0.05, and 0.45. Agreement was assessed with the compatibility index En = |D|/√(U_ref^2^ + U_method^2^), which folds in the uncertainty of the developed method as well as that of the assigned value; the values, computed from the unrounded uncertainties, were 0.25 for Li, 0.24 for Fe, 0.01 for P, and 0.49 for Ti (Figure 6b, Table 5). All were well below unity, providing independent evidence that the selected microwave digestion–ICP-OES procedure did not introduce a practically significant bias.

A secondary plausibility check used the independently assigned carbon value of 1.4846%. Because the purpose of this check is to test the developed method, the method means were used as the input. The five quantified components summed to 58.62%, leaving 41.38% attributable mainly to oxygen, close to the theoretical oxygen fraction of 40.57% in LiFePO_4_. The molar ratios were Li/P = 0.991 and (Fe + Ti)/P = 0.941. A residual Fe bias is an unlikely explanation for the latter: Fe recovered at 99.3% on the synthetic material, showed the smallest repeatability RSD of the four analytes at 0.49%, differed from its assigned value by only −0.72%, and had the smallest robust coefficient of variation among the participating laboratories, so a systematic Fe under-recovery of the required size is not compatible with these observations. Genuine non-stoichiometry on the Fe site, a bias in P—which carries both the largest expanded uncertainty and the largest analyst effect of the four analytes—and the assumption that Ti substitutes on the Fe site are all more plausible, and the available data do not distinguish them without additional phase analysis. The minimum sample intake at which homogeneity was demonstrated is 0.1 g for Li, Fe, P, and C and 0.2 g for Ti, which are the test portions used throughout this work, so the test portions adopted here are supported by the homogeneity data rather than chosen independently of them. Because the characterization relates to one commercial lot, the assigned values are an external check for this material and are not universal composition specifications for all Ti-doped LFP products.

### 2.10. Practical Considerations for Routine Use

The procedure is intended for compositional quality control rather than for production, but its practical characteristics bear on routine applicability. Each run occupies 150 min (30 min ramp, 60 min hold, 60 min cooling) and three runs can be completed in a working day. The rotor accommodates eight vessels, but the conditions validated here were established with four to six vessels per run, corresponding to 12–18 test portions per day; operation at full rotor load would require separate verification. Acid consumption is 8 mL per test portion, corresponding to 32–48 mL per run and about 100–150 mL of spent acid per day. Relative to the multi-acid treatments commonly applied to this class of material, the most consequential feature is that hydrofluoric acid is not required. In the interlaboratory comparison, the three laboratories that used HF-containing digestion returned Ti values showing no practically significant difference from those of the eleven that did not, although, as noted in Section 2.9, residual differences below approximately 4% could not be resolved by that comparison. The comparison therefore excludes a loss on the scale observed with aqua regia while leaving smaller differences open, and on that basis results of comparable quality appear to be obtainable without HF, with the corresponding reduction in operator hazard, in waste-treatment burden, and in attack on quartz and glass components. The principal limitations are the batch size imposed by the rotor, the need to manage vessel pressure, and the observation that test portions of 0.3 g occasionally left undigested material at the same acid volume.

No product specification for the Ti content of this material is publicly available, and the supplier’s internal release specification could not be disclosed. Fitness for purpose is therefore expressed as the narrowest specification the method can support. For a decision rule with a guard band equal to the expanded uncertainty [28], we adopted, as a conservative criterion of our own, a specification half-width of at least four times the expanded uncertainty, so that the guard band takes up no more than one quarter of the interval. With the routine-condition uncertainties (Table S31), this criterion is met for Ti by specifications of ±13.4% relative or wider, i.e., about ±0.019% absolute at the Ti content of the investigated material; the values for Li, Fe, and P are given in Table S35. The routine standard uncertainty for Ti (1.68%) is also smaller than the between-laboratory standard deviation of the characterization exercise (2.0%), and less than one third of the Thompson-modified Horwitz predicted reproducibility (5.37%). A laboratory adopting the method should compare these limits with its own product specification before using the results for release decisions.

On the basis of the validation data, the following routine quality-control measures are recommended for laboratories adopting the procedure. (i) Because digestion temperature is the critical control factor (Section 2.7), the temperature record of every run should be checked to confirm that 240 °C was reached and held for 60 min; a run that fails this check should be repeated rather than corrected. (ii) A procedural blank should be digested with every batch, and a mid-range calibration-verification solution should be measured before and after the samples, together with a 0.5 mg L^−1^ Ti check solution at the lower calibration limit (Table S33). (iii) A control sample of a well-characterized material of the same type, for example, the investigated lot with the assigned values in Table 5, should be digested with every batch and plotted on a control chart with warning and action limits of ±2 and ±3 intermediate-precision standard deviations (Table 3), i.e., ±2.8% and ±4.3% relative for Ti. (iv) Duplicate digestions should agree within the repeatability limit r = 2.8s_r_, corresponding to 4.2% (Li), 1.4% (Fe), 2.6% (P), and 1.8% (Ti) relative (Table S14). (v) Visual absence of residue should not be used as an acceptance criterion for Ti (Section 2.2), and participation in proficiency testing, where available, should provide the external check that a single laboratory cannot supply.

### 2.11. Scope and Limitations

The scope of this validation should be stated explicitly. The work was carried out in a single laboratory on a single commercial lot of carbon-coated Ti-doped LFP with D50 = 0.381 µm and a total carbon content of 1.4846%. Both the digestion temperature required for quantitative Ti release and the magnitude of dopant retention under aqua regia depend on the amount and nature of the surface carbon, so extrapolation to Ti-doped LFP products with different particle size or carbon content is not supported by these data and should be verified for each material. The assigned values used as an external check likewise relate to this lot and are not composition specifications for the product class.

## 3. Materials and Methods

### 3.1. Sample, Reagents, and Instrumentation

A commercial Ti-doped, carbon-coated LFP powder produced for lithium-ion battery cathode applications (LF-1BS; L&F Co., Ltd., Daegu, Republic of Korea) was used. The material was drawn from a single production lot. The lot is separately the subject of a reference-material characterization exercise, from which the property values in Section 2.9 are taken. The properties needed to interpret and reproduce this work are given below. The laser-diffraction D10, D50, and D90 values were 0.165, 0.381, and 1.63 μm, respectively. FE-SEM examination of 76 identifiable primary particles gave a number-based mean dimension of 0.283 ± 0.076 μm. Phase purity and crystallite size were examined by powder X-ray diffraction (PXRD), and particle morphology and elemental distribution by FE-SEM with EDS mapping (Figures S2–S4). All reflections in the PXRD pattern were indexed to orthorhombic olivine LiFePO_4_ (triphylite, space group Pnma; ICDD PDF 01-075-7725), and no reflection attributable to a crystalline impurity phase such as Li_3_PO_4_, Fe_2_P, an iron oxide, or TiO_2_ was observed. The apparent crystallite size and microstrain obtained by the Halder–Wagner method were about 64 nm and 0.05%. A separate Ti-bearing phase corresponding to 0.14% Ti could remain undetected by PXRD, so the diffraction data do not establish where the Ti is located. FE-SEM images (Figure S3) showed rounded primary particles, mostly 0.2–0.5 µm across, together with a smaller number of dense particles of 1–2 µm and loosely bound clusters of fine particles, consistent with the laser-diffraction size distribution. In the EDS maps of two areas (Figure S4), Fe, P, and O were co-distributed over the particles, with no apparent regions of selective enrichment. Local intensity variations followed the particle topography. Across the four maps obtained from two areas at two magnifications, the RSD of the map-sum mass fraction was no greater than 1.2% for each element, and the mean Fe/P atomic ratio was 1.05 (Table S36). These normalized, semi-quantitative results were used to compare areas and magnifications, rather than to assess agreement with the bulk composition measured by ICP-OES. Li could not be mapped because its characteristic X-ray energy (about 0.05 keV) lies below the low-energy limit of the EDS detector used, and Ti was not identified in the map-sum spectra, consistent with a content of about 0.14% lying at or below the EDS detection limit; Ti was, by contrast, detected in the aqua regia residue, in which it is enriched (Section 2.1). Homogeneity at the scale of the analytical test portion was established separately by the between-bottle homogeneity study summarized in Section 2.9; that study does not describe the micrometre-scale distribution of Li. The assigned mass fractions of the lot were 4.2913% Li, 33.301% Fe, 19.590% P, 0.14272% Ti, and 1.4846% C.

Electronic-grade nitric acid (CTPDA03) and hydrochloric acid (CTPDA08) were obtained from Chemitop (Jincheon, Republic of Korea). Water had a resistivity of at least 18.2 MΩ cm. NIST-traceable single-element standards for Li, Fe, P, Ti, and Y were supplied by Inorganic Ventures (Christiansburg, VA, USA). Working standards were prepared on the day of use. Polypropylene containers were used for solution storage.

Digestion was performed with an ETHOS EASY microwave system (Milestone, Sorisole, Italy). Elemental determinations were performed using an Avio 500 ICP-OES instrument (PerkinElmer, Shelton, CT, USA). Residues were examined using a JSM-7610 field-emission scanning electron microscope equipped with an energy-dispersive X-ray spectrometer (JEOL, Tokyo, Japan). PXRD patterns were recorded with a SmartLab diffractometer (Rigaku, Tokyo, Japan) operated at 40 kV and 200 mA with Cu Kα radiation (λ = 1.5406 Å) and a scintillation counter, over 2θ = 10–80° with a step size of 0.02°; phases were identified against the ICDD PDF-2 (2023) database, and the crystallite size and microstrain were evaluated by the Halder–Wagner method, in which (β/tan θ)^2^ is plotted against β/(tan θ sin θ) for the integral breadths β of the indexed reflections, the slope yielding the crystallite size and the intercept the microstrain. The starting material was examined with an SU5000 field-emission scanning electron microscope (Hitachi High-Tech, Tokyo, Japan) equipped with an Ultim Max silicon drift energy-dispersive X-ray detector (Oxford Instruments, High Wycombe, UK); the powder was fixed on carbon tape without conductive coating, images were recorded at 10 kV and EDS maps at 15 kV with a working distance of 20 mm, and C was excluded from EDS quantification because of the carbon-tape substrate. Particle size was measured using an LA-950 laser diffraction particle size analyzer (HORIBA, Kyoto, Japan).

### 3.2. Aqua Regia Applicability Test and Residue Analysis

The LFP was treated according to the aqua regia hot-plate concept used in KS L 1629-1 [10], following its trace-element conditions of a 0.5 g test portion diluted to 100 mL, with HCl:HNO_3_ = 3:1. After heating, the digest was cooled to room temperature, filtered, and the retained material was visually inspected. Dried residue was examined by FE-SEM/EDS at six locations. For mass-balance analysis, seven independent digestions were performed on each of two filter media, No. 5C quantitative cellulose filter paper (Advantec, Tokyo, Japan) and GF/F glass-fibre filters (Whatman, Maidstone, UK). Filters were rinsed with dilute hydrochloric acid and then with warm water until the filtrate was neutral, dried at 60 °C to constant mass, and weighed; the tare mass of each filter had been determined under the same drying conditions, and blank filters were carried through the identical sequence to correct the residue mass. A drying temperature of 60 °C was used because residual acid retained in cellulose filters causes discolouration and mass change at higher temperatures. The retained solids were then ashed in platinum crucibles, dissolved using HF and HCl, and evaporated to dryness before being taken up for measurement by ICP-OES. Analyte losses during ashing, evaporation, and transfer of the residue were not quantified, so the retained fractions are reported as measured values for this work-up; the same procedure was applied to every digestion in this series. Hydrofluoric acid was used only in this ancillary residue analysis; the digestion method evaluated in this work uses nitric and hydrochloric acid only. Residue mass fractions were expressed relative to the original test portion, and retention was calculated as the residue-derived analyte fraction divided by the total analyte content. The aqua regia filtrates were analyzed in parallel so that filtrate and residue were obtained from the same test portion.

### 3.3. Microwave Digestion Procedure

For Li, Fe, and P, approximately 0.1 g of sample was weighed into a microwave vessel. For Ti, 0.20 ± 0.01 g was used. HCl (2 mL) and HNO_3_ (6 mL) were added; the mixture was made up with water to 12 mL, the minimum liquid volume specified for the rotor so that all vessels heat uniformly, and the vessels were closed. Digestion was performed with an ETHOS EASY microwave system equipped with a direct temperature sensor; the applied microwave power is regulated automatically by the instrument to follow the programmed temperature profile and is therefore not an operator setting, the maximum available power being 1900 W (2 × 950 W). The vessels were held in a NOVA-8 high-temperature rotor (Milestone, Italy), in which HTV-75 TFM vessels (75 mL) are placed in ceramic safety shields and closed with spring-loaded pressure-calibration units; the rotor is rated to 300 °C and 100 bar (operating pressure 80 bar), with a limit of 260 °C and a hold of up to 2 h for HCl-containing acid mixtures such as the one used here. No maximum sample mass is specified by the manufacturer; at the acid volume used here, 0.2 g was the largest test portion that consistently gave residue-free digests (Section 2.2), and it is therefore the validated maximum load per vessel. The rotor accommodates eight vessels, and four to six vessels were processed per run. The temperature was ramped to 240 °C over 30 min, held for 60 min, and followed by 60 min cooling. Each digest was filtered through No. 5C quantitative cellulose filter paper; the Li/Fe/P digest was then diluted to 1000 mL. The Ti digest was diluted to 100 mL. Procedural blanks were processed with every batch. For the temperature study, otherwise identical preparations were digested at 220, 230, 235, 240, and 245 °C, with seven replicates per condition and the vessel load held constant across conditions. Here, “microwave digestion” denotes acid decomposition in sealed vessels in which the microwave field serves only to heat the acid mixture by dielectric heating; no microwave discharge or plasma is generated in the vessel, so the process is distinct from the microwave-discharge and plasma processes used, for example, in the synthesis of metal–carbon nanostructures.

### 3.4. ICP-OES Measurement and Calibration

The ICP-OES operating conditions are listed in Table S32. Measurements were made on a single ICP-OES instrument throughout, so the intermediate-precision results in Section 2.5 reflect analyst and run effects only. Argon was used for the plasma, auxiliary, and nebulizer flows, for the sealed optical purge of the spectrometer, and as the shear gas; the purge gas was supplied at a regulated pressure of 0.4–0.5 MPa, within the range specified by the instrument manufacturer, and the optical purge was operated at its normal flow setting, the flow itself being managed by the instrument rather than set by the operator; measurements at P 178.221 nm were made after the purge had stabilized. Radial viewing was used for Li 670.784, Fe 259.939, and Ti 334.940 nm, and axial viewing for P 178.221 nm, which was selected because the vacuum-UV line gives a markedly lower response than the other lines; all four candidate P wavelengths were evaluated under axial viewing, so the wavelength selection was not confounded by viewing mode, and background correction and integration time were identical for both viewing modes. Two-point background correction was applied to every analytical line, with no interelement or multicomponent spectral fitting, and signals were integrated as peak area over three points per peak. The read time was automatic within a 1–5 s window with a 60 s read delay. The plasma was stabilized, and wavelength calibration and instrument-performance checks were completed before measurement. Calibration solutions covered the working ranges stated in Section 2.4, and calibration standards, blanks, and the internal standard solution were matched to the acid concentration of the corresponding sample solutions. Each solution (calibration standards and samples alike) was measured with three replicate readings, and the mean was reported. Intensities were corrected against Y at 371.029 nm and blank-subtracted. A calibration-verification solution was measured before and after sample measurements. Samples were prepared independently, and the reported result was the mean of replicate preparations. The temperature study and the aqua regia comparison were measured in separate sessions; to link them, three digests from the 240 °C level of the temperature study were re-measured within the aqua regia session against that session’s calibration, and the means agreed with the original session to within −0.01% (Li), +0.05% (Fe), −0.34% (P), and +0.45% (Ti), none of the differences being significant (*p* = 0.57–0.98).

### 3.5. Validation Design and Statistical Treatment

The validation design followed KOLAS-G-009 and the general principles of ISO/IEC 17025, ISO 5725-2, the Eurachem method-validation guide, and AOAC performance guidance [11,12,13,14,15]. Selectivity was evaluated as described in Section 2.3. Linearity was assessed by least-squares regression together with concentration-normalized residuals. Sensitivity was represented by the calibration slope and its between-curve RSD.

For major-element trueness, seven independently prepared synthetic mixtures were measured against theoretical composition. Ti recovery was evaluated by post-digestion additions at 0.05%, 0.15%, and 0.25% on a sample basis, with seven independent preparations per level. Repeatability comprised seven complete analyses by one analyst. Intermediate precision comprised three analysts × seven complete analyses. Analyst effects were assessed by conventional one-way ANOVA and, as a sensitivity analysis where the equal-variance assumption was questionable, by Welch ANOVA [29]. Precision criteria were informed by the Thompson-modified Horwitz relationship [30,31].

All statistical calculations were performed using Microsoft Excel 2019 (Microsoft Corp., Redmond, WA, USA); one-way analysis of variance was computed with the Analysis ToolPak, and Welch analysis of variance was implemented from its defining formulae. Intermediate-precision estimates were derived from the analysis-of-variance components rather than from the pooled standard deviation of all determinations.

### 3.6. Uncertainty Evaluation

Measurement uncertainty was evaluated according to the GUM framework [32]. The measurement model included test-portion mass, final volume, calibration-derived concentration, and repeatability. Type B standard uncertainties for mass and volume were obtained from calibration certificates and tolerances; calibration uncertainty was obtained from the regression prediction standard deviation; repeatability uncertainty was calculated as s/√n. Components were propagated using sensitivity coefficients and combined by the root-sum-of-squares method. Expanded uncertainty was calculated using k = 2, corresponding to an approximate 95% level of confidence.

### 3.7. Interlaboratory External Verification

The same commercial material was distributed to qualified laboratories as part of a broader candidate-reference-material characterization study. Laboratory means were screened using Grubbs’ test, assessed for distributional behaviour, and summarized using ISO 13528, ISO 17034, and ISO Guide 35 principles [26,33,34]. The present method was not used to establish the comparison values; its mean was compared post hoc with the multi-laboratory assigned value and expanded uncertainty. Digestion-method subgroup means were also compared to examine whether the selected microwave procedure differed systematically from HF-containing multi-acid approaches.

## 4. Conclusions

Aqua regia hot-plate digestion was not suitable for quantitative Ti determination in the investigated carbon-coated, Ti-doped LFP: 5.86 ± 0.37% of total Ti remained in a carbon-dominated residue, whereas only 0.39–0.81% of Li, Fe, and P was retained, and direct analysis of the filtrates returned 92.8% of the assigned Ti value against 100.0% for the microwave procedure. A closed-vessel procedure using 6 mL HNO_3_ and 2 mL HCl with a 240 °C set point produced quantitative Ti release; between 220 and 240 °C the Ti result rose by 14.0% while the major elements changed by less than 2%, with the transition between 230 and 235 °C and a plateau at and above 240 °C. Matrix-selective wavelengths, calibration R^2^ values above 0.999, recoveries near 100%, and repeatability RSDs below 1.6% were obtained. Conventional ANOVA did not identify significant analyst effects, although Welch analysis placed Fe at *p* = 0.047 and P at *p* = 0.052; the corresponding analyst-mean differences remained within the practical acceptance intervals. The lower calibration limit for Ti was 0.5 mg L^−1^, equivalent to 250 mg kg^−1^ on a solid basis. At this level, the matrix-matched check solution gave a Ti recovery of 99.9%, and the model-based relative expanded uncertainty (k = 2) for a single result was approximately 9% under single-day conditions. At the concentrations found in the investigated material, the relative expanded uncertainties of the reported method means ranged from 0.64% to 2.20%. Agreement with multi-laboratory assigned values, expressed as compatibility indices well below unity, provided an external trueness check. Post-digestion spike recovery was interpreted only as evidence for the measurement stage, while digestion completeness was supported separately by residue mass balance and interlaboratory comparison. The method is fit for compositional quality control of the investigated Ti-doped LFP material where the Ti specification is ±13.4% relative or wider (half-width at least four times the routine expanded uncertainty), provided that the 240 °C set point and specified test-portion-to-acid ratios are maintained and that each batch is supported by a check of the temperature record, a procedural blank, calibration and lower-limit verification, and a control sample; because validation was carried out in a single laboratory on a single commercial lot, substantially different products or digestion systems require laboratory verification.

## Figures and Tables

**Table 1:** Aqua regia and closed-vessel microwave digestion compared on the same lot. Filtrate results are expressed as recovery against the interlaboratory assigned value; the retained fraction is from the residue mass balance of the 5C set; microwave values are from the 240 °C set of the temperature study (Section 2.7, Table S25). 5C, No. 5C quantitative cellulose filter paper; GF/F, glass-fibre filter. The microwave digests were filtered through No. 5C filter paper.

Analyte	Assigned Value (%)	Aqua Regia, 5C (n = 7)	Aqua Regia, GF/F (n = 7)	Aqua Regia, Pooled (n = 14)	Retained in Residue (%)	Microwave, 240 °C (n = 7)
Li	4.2913	100.24%	100.90%	100.57%	0.39 ± 0.12	100.4%
Fe	33.3011	97.60%	98.27%	97.94%	0.53 ± 0.11	99.9%
P	19.5900	99.10%	100.18%	99.64%	0.81 ± 0.13	100.0%
Ti	0.142716	92.11%	93.45%	92.78%	5.86 ± 0.37	100.0%

**Table 2:** Calibration performance.

Element	Working Range (mg L^−1^)	Expected Sample Solution (mg L^−1^)	R^2^	Residual Near Sample (%)	Slope RSD (%)
Li	1–7	4.29	0.999771	+1.1	0.23
Fe	5–60	33.30	0.999804	−1.6	0.08
P	5–35	19.59	0.999037	−1.0	0.62
Ti	0.5–5	2.85	0.999814	−1.3	0.13

**Table 3:** Trueness and precision summary.

Element	Recovery (%)	Sample Mean (%)	Repeatability RSD (%)	Intermediate Precision RSD (%)	ANOVA *p*	Predicted Reproducibility RSD (%)
Li	99.8	4.3505	1.51	1.57	0.144	3.21
Fe	99.3	33.062	0.49	0.56	0.062	1.74
P	99.0	19.582	0.94	2.02	0.141	2.26
Ti	98.6–99.6	0.14073	0.64	1.42	0.880	5.37

**Table 4:** Measurement results and expanded uncertainties.

Element	Result (Mass Fraction, %)	Expanded Uncertainty, U (%, k = 2)	Relative U (%)	Dominant Component
Li	4.35	0.058	1.34	Repeatability (72.9%)
Fe	33.06	0.213	0.64	Calibration (47.6%)
P	19.58	0.431	2.20	Calibration (87.9%)
Ti	0.141	0.0021	1.49	Calibration (84.8%)

**Table 5:** Comparison with property values assigned by interlaboratory characterization.

Element	Method Mean (%)	Assigned Value (%)	Assigned U (%, k = 2)	Relative Difference (%)	En
Li	4.3505	4.2913	0.227	+1.38	0.25
Fe	33.062	33.301	0.966	−0.72	0.24
P	19.582	19.590	0.909	−0.04	0.01
Ti	0.14073	0.14272	0.0034	−1.39	0.49

## Data Availability

All data used to support the findings of this study are included in the article and Supplementary Materials, with the exception of the full homogeneity and stability datasets of the candidate reference material; these are summarized in Table S29 and are available from the corresponding author on reasonable request. Additional instrument-output files are available from the corresponding author on reasonable request.

## Supplementary Materials

The following supporting information can be downloaded at: https://www.mdpi.com/article/10.3390/molecules31213915/s1, Figure S1, showing the appearance of the digests obtained in the temperature study; Figures S2–S4, showing the PXRD pattern, FE-SEM images, and EDS elemental maps of the investigated material; and Tables S1–S36, containing the residue EDS analysis and mass balance; complete selectivity data for all sixteen candidate wavelengths and the associated matrix-bias regression; calibration data; individual trueness, recovery, repeatability, and analyst data; a Ti sensitivity check measured below the lower calibration limit; uncertainty budgets for the method and for the assigned property values; the temperature-dependence dataset; the aqua regia filtrate, residue mass and loss-on-ignition results; the linking of the two measurement sessions; external-verification results, including the coded laboratory-level results of the interlaboratory characterization exercise; methodological notes required to interpret post-digestion spike recovery and analyst-effect testing; the ICP-OES operating conditions; the verification and uncertainty of Ti results at the lower calibration limit; and the specification limits supported by the method uncertainty.
