# Supplementary material for: Overcoming Incomplete Digestion of Ti-Doped LiFePO4 Cathode Materials: Microwave-Assisted Sample Preparation and Single-Laboratory Validated ICP-OES Determination of Li, Fe, P, and Ti
Source: Molecules. 2026 Oct 30;31(21):3915. doi: 10.3390/molecules31213915 (PMC13765147; doi:10.3390/molecules31213915)
Supplement: Supplementary file 1 [file molecules-31-03915-s001.zip › molecules-4610220-supplementary.pdf]

# Supplementary Information

## Overcoming Incomplete Digestion of Ti-Doped $LiFePO_4$ Cathode Materials: Microwave-Assisted Sample Preparation and Single-Laboratory Validated ICP-OES Determination of Li, Fe, P, and Ti

Myung-Gyu Lee, Tae-Young Kim, Sam-Hang Yi, Joo-Seok Park, Young-Jun Yoon and Kyoung-Seok Moon

This Supplementary Information provides the supporting numerical datasets used for the validation and external verification reported in the main manuscript. It includes residue characterization and mass balance, selectivity, calibration, trueness, recovery, repeatability, intermediate-precision, sensitivity, uncertainty and interlaboratory-comparison data, together with the temperature-dependence dataset, the aqua-regia filtrate and residue results, the uncertainty budget of the assigned property values, the routine-conditions uncertainty and the linking of the two measurement sessions. Section S9 gives the ICP-OES operating conditions, the verification and uncertainty of Ti results at the lower calibration limit, the specification limits supported by the method uncertainty, and the PXRD, FE-SEM and EDS characterization of the investigated material (LF-1BS; L&F Co., Ltd., Daegu, Republic of Korea).

## S1. Residue Characterization after Aqua-Regia Digestion

**Table S1.** EDS analysis of the undissolved residue recovered after open-vessel aqua-regia digestion (six observation points; mass fraction, %).

| Point | C | O | Al | Si | P | Cl | Ti | Fe | Zr | Total | Fe/P (at.) |
|---|---|---|---|---|---|---|---|---|---|---|---|
| 1 | 73.14 | 15.64 | 0.47 | 0.15 | 2.77 | 6.27 | 0.23 | 1.34 | – | 100 | 0.27 |
| 2 | 72.75 | 13.66 | 0.67 | 0.11 | 3.09 | 7.55 | 0.29 | 1.88 | – | 100 | 0.34 |
| 3 | 75.54 | 12.79 | 0.49 | 0.07 | 3.23 | 6.04 | 0.27 | 1.57 | – | 100 | 0.27 |
| 4 | 68.12 | 12.93 | 0.38 | 0.25 | 4.75 | 7.95 | 0.34 | 1.89 | 3.39 | 100 | 0.22 |
| 5 | 75.10 | 11.36 | 0.50 | – | 4.07 | 6.59 | 0.41 | 1.97 | – | 100 | 0.27 |
| 6 | 73.52 | 16.43 | 0.29 | 0.27 | 3.19 | 4.76 | 0.24 | 1.30 | – | 100 | 0.23 |
| Mean | 73.03 | 13.80 | 0.47 | 0.17 | 3.52 | 6.53 | 0.30 | 1.66 | – | – | 0.27 |
| SD | 2.65 | 1.90 | 0.13 | 0.09 | 0.74 | 1.14 | 0.07 | 0.30 | – | – | 0.04 |

*Values are normalised to 100%. Ti was detected at every observation point. Zr was detected only at point 4, indicating local heterogeneity of the residue. EDS is semi-quantitative and does not detect Li; the Fe/P ratio is given on an atomic basis after conversion from the mass fractions.*

**Table S2. Mass balance of the aqua-regia residue, seven independent digestions (mean values).**

| Element | Residue content (%) | Total content in sample (%) | Fraction of total analyte retained (%) | Enrichment relative to Fe |
|---|---|---|---|---|
| Li | 0.01660 | 4.291 | 0.39 | 0.7 |
| Fe | 0.17655 | 33.301 | 0.53 | 1.0 |
| P | 0.15924 | 19.590 | 0.81 | 1.5 |
| Ti | 0.00836 | 0.1427 | 5.86 | 11.0 |

*Mean of seven independent digestions on quantitative cellulose filters, expressed on the basis of the original test portion. Standard deviations of the retained fractions were 0.12% (Li), 0.11% (Fe), 0.13% (P) and 0.37% (Ti); individual values are given in Table S27. The total contents used as denominators are the interlaboratory assigned values. Together with Table S1 these data establish that the dopant, not the major elements, is selectively retained when bulk dissolution appears visually complete. Possible analyte losses during the residue work-up (ashing, evaporation to dryness and transfer) were not quantified, and the retained fractions are reported as measured.*

## S2. Selectivity Data

**Table S3.** Measured Li concentration (mg L⁻¹) at each coexisting-element level for four candidate wavelengths. The Li concentration was held at 10 mg L⁻¹ and Fe, P were varied.

| Wavelength (nm) | 0 mg L⁻¹ | 10 mg L⁻¹ | 20 mg L⁻¹ | 50 mg L⁻¹ | 100 mg L⁻¹ | Mean | SD | RSD (%) |
|---|---|---|---|---|---|---|---|---|
| 670.784 | 10.08 | 10.05 | 10.08 | 10.05 | 10.09 | 10.07 | 0.019 | 0.19 |
| 610.362 | 9.89 | 10.12 | 10.18 | 9.99 | 10.16 | 10.07 | 0.123 | 1.23 |
| 460.286 | 8.96 | 8.97 | 10.30 | 13.34 | 13.15 | 10.94 | 2.170 | 19.83 |
| 413.256 | 12.98 | 14.92 | 15.24 | 15.73 | 24.17 | 16.61 | 4.354 | 26.22 |

**Table S4.** Measured Fe concentration (mg L⁻¹) at each coexisting-element level for four candidate wavelengths. The Fe concentration was held at 10 mg L⁻¹ and Li, P were varied.

| Wavelength (nm) | 0 mg L⁻¹ | 10 mg L⁻¹ | 20 mg L⁻¹ | 50 mg L⁻¹ | 100 mg L⁻¹ | Mean | SD | RSD (%) |
|---|---|---|---|---|---|---|---|---|
| 238.204 | 10.03 | 10.01 | 10.07 | 10.06 | 10.05 | 10.04 | 0.024 | 0.24 |
| 239.562 | 10.03 | 9.97 | 10.03 | 9.98 | 10.01 | 10.00 | 0.027 | 0.27 |
| 259.939 | 10.04 | 10.02 | 10.06 | 10.04 | 10.02 | 10.04 | 0.017 | 0.17 |
| 234.349 | 10.08 | 10.10 | 10.13 | 10.39 | 10.63 | 10.27 | 0.239 | 2.33 |

**Table S5.** Measured P concentration (mg L⁻¹) at each coexisting-element level for four candidate wavelengths. The P concentration was held at 10 mg L⁻¹ and Li, Fe were varied.

| Wavelength (nm) | 0 mg L⁻¹ | 10 mg L⁻¹ | 20 mg L⁻¹ | 50 mg L⁻¹ | 100 mg L⁻¹ | Mean | SD | RSD (%) |
|---|---|---|---|---|---|---|---|---|
| 213.617 | 10.05 | 10.06 | 10.03 | 10.03 | 9.97 | 10.03 | 0.035 | 0.35 |
| 214.914 | 9.92 | 10.01 | 10.01 | 10.04 | 9.98 | 9.99 | 0.045 | 0.46 |
| 178.221 | 10.05 | 10.00 | 9.99 | 10.04 | 10.01 | 10.02 | 0.026 | 0.26 |
| 177.434 | 9.89 | 9.72 | 9.85 | 9.73 | 9.95 | 9.83 | 0.102 | 1.03 |

**Table S6.** Measured Ti concentration (mg L⁻¹) at each coexisting-element level for four candidate wavelengths. The Ti concentration was held at 10 mg L⁻¹ and Li, Fe, P were varied.

| Wavelength (nm) | 0 mg L⁻¹ | 100 mg L⁻¹ | 200 mg L⁻¹ | 500 mg L⁻¹ | 1000 mg L⁻¹ | Mean | SD | RSD (%) |
|---|---|---|---|---|---|---|---|---|
| 334.940 | 10.02 | 10.00 | 9.99 | 9.98 | 9.96 | 9.99 | 0.022 | 0.23 |
| 336.121 | 9.89 | 9.96 | 9.97 | 9.88 | 9.98 | 9.94 | 0.049 | 0.49 |
| 337.279 | 9.82 | 9.92 | 9.99 | 9.80 | 9.84 | 9.87 | 0.079 | 0.80 |
| 334.903 | 9.86 | 9.89 | 10.05 | 9.76 | 9.83 | 9.88 | 0.106 | 1.07 |

**Table S7.** Regression of recovery on coexisting-element concentration for all sixteen candidate wavelengths.

| Element | λ (nm) | Repeatability RSD (%) | Recovery range (%) | Max. deviation (%) | Slope | Bias at max. matrix (%) | 95% CI of bias (%) |
|---|---|---|---|---|---|---|---|
| Li | 670.784 | 0.197 | 100.46–100.89 | 0.89 | $1.77\times10^{-3}$ | +0.18 | −0.22 to +0.57 |
| Li | 610.362 | 0.446 | 98.90–101.82 | 1.82 | $1.20\times10^{-2}$ | +1.20 | −0.63 to +3.04 |
| Li | 460.286 | 0.373 | 89.64–133.40 | 33.40 | $4.72\times10^{-1}$ | +47.22 | +31.79 to +62.65 |
| Li | 413.256 | 0.172 | 129.81–241.68 | 141.68 | 1.02 | +102.45 | +82.28 to +122.62 |
| Fe | 238.204 | 0.198 | 100.07–100.70 | 0.70 | $2.52\times10^{-3}$ | +0.25 | −0.21 to +0.72 |
| Fe | 239.562 | 0.385 | 99.72–100.30 | 0.30 | $-2.73\times10^{-4}$ | −0.03 | −0.68 to +0.62 |
| Fe | 259.939 | 0.243 | 100.20–100.60 | 0.60 | $-1.30\times10^{-3}$ | −0.13 | −0.53 to +0.27 |
| Fe | 234.349 | 0.444 | 100.82–106.32 | 6.32 | $5.87\times10^{-2}$ | +5.87 | +5.06 to +6.67 |
| P | 213.617 | 0.279 | 99.71–100.60 | 0.60 | $-8.14\times10^{-3}$ | −0.81 | −1.24 to −0.39 |
| P | 214.914 | 0.416 | 99.17–100.41 | 0.83 | $2.54\times10^{-3}$ | +0.25 | −0.64 to +1.14 |

| Element | λ (nm) | Repeatability RSD (%) | Recovery range (%) | Max. deviation (%) | Slope | Bias at max. matrix (%) | 95% CI of bias (%) |
|---|---|---|---|---|---|---|---|
| P | 178.221 | 0.212 | 99.90–100.47 | 0.47 | $-8.79\times10^{-4}$ | −0.09 | −0.57 to +0.39 |
| P | 177.434 | 0.446 | 97.18–99.52 | 2.82 | $1.06\times10^{-2}$ | +1.06 | −0.45 to +2.57 |
| Ti | 334.940 | 0.241 | 99.56–100.22 | 0.44 | $-5.38\times10^{-4}$ | −0.54 | −0.90 to −0.18 |
| Ti | 336.121 | 0.396 | 98.75–99.83 | 1.25 | $3.68\times10^{-4}$ | +0.37 | −0.51 to +1.25 |
| Ti | 337.279 | 0.210 | 97.97–99.91 | 2.03 | $-6.47\times10^{-4}$ | −0.65 | −1.80 to +0.51 |
| Ti | 334.903 | 0.310 | 97.60–100.45 | 2.40 | $-1.11\times10^{-3}$ | −1.11 | −2.60 to +0.37 |

*Slope units are % recovery per mg $L^{-1}$ of coexisting element. Wavelengths were selected on the basis of both the recovery interval and the confidence interval of the matrix-dependent bias, rather than on sensitivity alone. Li 460.286 and 413.256 nm show severe positive matrix-dependent deviation and Fe 234.349 nm rises to 106.3%, whereas P 177.434 nm shows a persistent negative deviation. The selected lines were Li 670.784, Fe 259.939, P 178.221 and Ti 334.940 nm.*

## S3. Calibration Data

### Table S8. Calibration levels and internal-standard-corrected intensities for Li ($R^2$ = 0.999771).

| Concentration (mg $L^{-1}$) | Reading 1 | Reading 2 | Reading 3 | Mean intensity |
|---|---|---|---|---|
| 1 | 115,194 | 116,105 | 115,575 | 115,624 |
| 2 | 218,756 | 218,252 | 218,049 | 218,352 |
| 3 | 310,321 | 313,835 | 312,362 | 312,173 |
| 4 | 414,032 | 413,685 | 416,224 | 414,647 |
| 5 | 504,078 | 508,264 | 508,429 | 506,924 |
| 6 | 602,694 | 597,949 | 603,457 | 601,367 |
| 7 | 697,972 | 703,333 | 700,688 | 700,664 |

### Table S9. Calibration levels and internal-standard-corrected intensities for Fe ($R^2$ = 0.999804).

| Concentration (mg $L^{-1}$) | Reading 1 | Reading 2 | Reading 3 | Mean intensity |
|---|---|---|---|---|
| 5 | 77,353 | 77,342 | 77,273 | 77,323 |
| 10 | 159,895 | 160,158 | 160,336 | 160,130 |
| 20 | 322,233 | 322,728 | 322,612 | 322,524 |
| 30 | 480,056 | 480,256 | 480,752 | 480,355 |
| 40 | 657,339 | 660,942 | 659,633 | 659,305 |
| 50 | 813,677 | 812,825 | 814,652 | 813,718 |
| 60 | 985,636 | 987,034 | 984,009 | 985,560 |

### Table S10. Calibration levels and internal-standard-corrected intensities for P ($R^2$ = 0.999037).

| Concentration (mg $L^{-1}$) | Reading 1 | Reading 2 | Reading 3 | Mean intensity |
|---|---|---|---|---|
| 5 | 289 | 304 | 293 | 295 |
| 10 | 609 | 579 | 594 | 594 |
| 15 | 909 | 903 | 923 | 912 |
| 20 | 1,215 | 1,225 | 1,231 | 1,224 |
| 25 | 1,524 | 1,541 | 1,569 | 1,545 |
| 30 | 1,914 | 1,862 | 1,903 | 1,893 |
| 35 | 2,190 | 2,228 | 2,159 | 2,192 |

**Table S11. Calibration levels and internal-standard-corrected intensities for Ti ($R^2$ = 0.999814; the 0.1 mg $L^{-1}$ level was excluded from the final regression).**

| Concentration (mg $L^{-1}$) | Reading 1 | Reading 2 | Reading 3 | Mean intensity |
|---|---|---|---|---|
| 0.1 * | 3,837 | 3,997 | 3,976 | 3,937 |
| 0.5 | 19,321 | 19,041 | 19,095 | 19,152 |
| 1.0 | 39,392 | 39,146 | 39,505 | 39,347 |
| 2.0 | 79,560 | 79,580 | 79,368 | 79,503 |
| 3.0 | 117,678 | 118,463 | 117,325 | 117,822 |
| 4.0 | 160,856 | 161,009 | 160,592 | 160,819 |
| 5.0 | 199,410 | 199,333 | 199,406 | 199,383 |

** Excluded from the final regression because it lies +29.8% above the extrapolated calibration (individual calibrations +22.5% to +34.3%). The working range for Ti was therefore 0.5–5.0 mg $L^{-1}$. Each reading is a consecutive instrument reading of the same calibration solution; all intensities were corrected with the Y internal standard at 5 mg $L^{-1}$ and blank-subtracted. The blank-corrected origin is not treated as a calibration point, since its value is fixed at zero by the blank correction and it carries no independent information. Regressions were therefore fitted over the measured standards alone; mean slopes were 96,996 (Li), 16,488 (Fe), 63.73 (P) and 40,122 (Ti) with RSDs of 0.23%, 0.08%, 0.62% and 0.13% across three independently prepared calibrations, and the corresponding intercepts were +21,979, −6,582, −38.3 and −977 counts, equivalent to +19.0%, −8.5%, −13.0% and −5.1% of the lowest standard of each range.*

## S4. Trueness and Recovery Data

**Table S12.** Trueness of Li, Fe, and P using the laboratory-prepared material (n = 7).

| No. | Li (%) | Fe (%) | P (%) |
|---|---|---|---|
| 1 | 4.41 | 34.79 | 19.79 |
| 2 | 4.41 | 34.81 | 19.04 |
| 3 | 4.37 | 34.88 | 19.45 |
| 4 | 4.38 | 35.49 | 19.21 |
| 5 | 4.34 | 35.08 | 19.37 |
| 6 | 4.42 | 35.55 | 19.79 |
| 7 | 4.39 | 35.47 | 19.42 |
| Mean | 4.39 | 35.15 | 19.44 |
| SD | 0.029 | 0.342 | 0.281 |
| RSD (%) | 0.65 | 0.97 | 1.45 |
| Reference (%) | 4.40 | 35.40 | 19.63 |
| Recovery (%) | 99.8 | 99.3 | 99.0 |
| Bias (%) | −0.2 | −0.7 | −1.0 |
| Limit (%) | ±3 | ±2 | ±2 |

*The material was prepared from high-purity $Li_2O$, $Fe_2O_3$ and $P_2O_5$. It reproduces the elemental composition of the sample but not the $LiFePO_4$ crystal phase or the carbon coating. Individual values are tabulated to two decimal places, whereas the summary statistics were computed from the unrounded results; recalculation from the tabulated values therefore reproduces the means but not the standard deviations exactly.*

**Table S13.** Individual Ti post-digestion spike-recovery results (n = 7 per level). Found values are in mg $L^{-1}$ and content values are mass fractions (%).

| No. | 0.05% found | 0.05% content | 0.15% found | 0.15% content | 0.25% found | 0.25% content |
|---|---|---|---|---|---|---|
| 1 | 0.988 | 0.0494 | 2.957 | 0.1473 | 4.976 | 0.2487 |
| 2 | 0.985 | 0.0492 | 2.988 | 0.1493 | 4.997 | 0.2512 |
| 3 | 0.985 | 0.0494 | 2.964 | 0.1471 | 4.881 | 0.2476 |
| 4 | 0.981 | 0.0490 | 2.997 | 0.1502 | 4.947 | 0.2456 |

| No. | 0.05% found | 0.05% content | 0.15% found | 0.15% content | 0.25% found | 0.25% content |
|---|---|---|---|---|---|---|
| 5 | 0.981 | 0.0490 | 2.976 | 0.1487 | 4.937 | 0.2459 |
| 6 | 0.988 | 0.0493 | 3.027 | 0.1501 | 4.988 | 0.2490 |
| 7 | 0.995 | 0.0498 | 3.011 | 0.1497 | 4.959 | 0.2478 |
| Mean | 0.9861 | 0.04929 | 2.9886 | 0.14892 | 4.9550 | 0.24799 |
| SD | 0.0048 | 0.00027 | 0.0252 | 0.00129 | 0.0391 | 0.00193 |
| RSD (%) | 0.49 | 0.55 | 0.84 | 0.87 | 0.79 | 0.78 |
| Recovery (%) | 98.6 | 98.6 | 99.6 | 99.6 | 99.1 | 99.1 |
| Bias (%) | −1.4 | −1.4 | −0.4 | −0.4 | −0.9 | −0.9 |

*Interpretive note for Table S13. Ti was added to the digest of the laboratory-prepared Li–Fe–P material, not to a digest of the commercial sample; that matrix contained Li, Fe and P at approximately 88, 708 and 393 mg $L^{-1}$ and no titanium, so the values tabulated correspond to the added amount and no correction for a native contribution was required. The recovery experiment therefore verifies the post-digestion measurement sequence (dilution, internal-standard correction, calibration and matrix response) in a matrix equivalent to that of a real Ti sample solution, but does not by itself demonstrate dissolution of Ti bound in the LFP/carbon-coated solid. Digestion completeness is supported separately by the residue mass balance (Tables S1, S2 and S27) and the interlaboratory comparison.*

## S5. Repeatability and Intermediate Precision

**Table S14.** Complete-method repeatability (n = 7).

| No. | Li (%) | Fe (%) | P (%) | Ti (%) |
|---|---|---|---|---|
| 1 | 4.38 | 33.12 | 19.58 | 0.1395 |
| 2 | 4.36 | 32.93 | 19.79 | 0.1415 |
| 3 | 4.38 | 33.06 | 19.52 | 0.1415 |
| 4 | 4.34 | 33.21 | 19.66 | 0.1417 |
| 5 | 4.21 | 32.82 | 19.79 | 0.1409 |
| 6 | 4.40 | 33.28 | 19.41 | 0.1403 |
| 7 | 4.38 | 33.01 | 19.31 | 0.1397 |
| Mean | 4.35 | 33.06 | 19.58 | 0.1407 |
| SD | 0.066 | 0.161 | 0.184 | 0.00090 |
| RSD (%) | 1.51 | 0.49 | 0.94 | 0.64 |
| $PRSD_r$ (%) | 1.60 | 0.87 | 1.13 | 2.69 |
| $HorRat_r$ | 0.94 | 0.56 | 0.83 | 0.24 |

*PRSD_r was taken as 0.5 × PRSD_R throughout; guides use either 0.5 or 0.66, and the choice propagates to every HorRat value in this table. The HorRat of 0.24 obtained for Ti falls below the customary 0.5–2.0 window; this may indicate repeatability that is optimistic relative to interlaboratory experience, or that the Horwitz relationship does not apply at this mass fraction and in this matrix, and this result is therefore reported with caution.*

**Table S15.** Intermediate-precision results for Li obtained by three analysts (mass fraction, %).

| No. | Analyst A | Analyst B | Analyst C |
|---|---|---|---|
| 1 | 4.3897 | 4.3633 | 4.4621 |
| 2 | 4.4057 | 4.4138 | 4.3077 |
| 3 | 4.3274 | 4.3189 | 4.3113 |
| 4 | 4.3631 | 4.4658 | 4.4506 |
| 5 | 4.3964 | 4.4822 | 4.4118 |
| 6 | 4.2363 | 4.4970 | 4.4405 |
| 7 | 4.3822 | 4.4561 | 4.3559 |
| Mean | 4.3573 | 4.4282 | 4.3914 |
| SD | 0.0593 | 0.0661 | 0.0658 |
| RSD (%) | 1.36 | 1.49 | 1.50 |

**Table S16.** Intermediate-precision results for Fe obtained by three analysts (mass fraction, %).

| No. | Analyst A | Analyst B | Analyst C |
|---|---|---|---|
| 1 | 33.1809 | 33.0010 | 32.9072 |
| 2 | 33.1429 | 33.2602 | 32.7404 |
| 3 | 32.6246 | 32.8805 | 32.6916 |
| 4 | 33.0159 | 32.9534 | 32.9249 |
| 5 | 33.1773 | 32.9406 | 32.6961 |
| 6 | 32.8722 | 33.1014 | 32.8024 |
| 7 | 33.1287 | 32.8529 | 32.9884 |
| Mean | 33.0204 | 32.9986 | 32.8216 |
| SD | 0.2065 | 0.1412 | 0.1193 |
| RSD (%) | 0.63 | 0.43 | 0.36 |

**Table S17.** Intermediate-precision results for P obtained by three analysts (mass fraction, %).

| No. | Analyst A | Analyst B | Analyst C |
|---|---|---|---|
| 1 | 19.7117 | 19.4867 | 20.3030 |
| 2 | 19.7333 | 20.1196 | 19.6635 |
| 3 | 19.7178 | 19.4990 | 20.0766 |
| 4 | 19.6825 | 19.8216 | 20.3953 |
| 5 | 19.8705 | 20.5545 | 19.5686 |
| 6 | 19.3564 | 20.8847 | 20.0590 |
| 7 | 19.8020 | 19.9408 | 20.4255 |
| Mean | 19.6963 | 20.0438 | 20.0702 |
| SD | 0.1629 | 0.5229 | 0.3424 |
| RSD (%) | 0.83 | 2.61 | 1.71 |

**Table S18.** Intermediate-precision results for Ti obtained by three analysts (mass fraction, %).

| No. | Analyst A | Analyst B | Analyst C |
|---|---|---|---|
| 1 | 0.13988 | 0.13916 | 0.14048 |
| 2 | 0.14545 | 0.14340 | 0.13799 |
| 3 | 0.13997 | 0.13917 | 0.14101 |
| 4 | 0.13899 | 0.13846 | 0.14205 |
| 5 | 0.13995 | 0.14264 | 0.14256 |
| 6 | 0.13891 | 0.13920 | 0.14163 |
| 7 | 0.13979 | 0.13893 | 0.13903 |
| Mean | 0.14042 | 0.14014 | 0.14068 |
| SD | 0.00226 | 0.00200 | 0.00165 |
| RSD (%) | 1.61 | 1.43 | 1.18 |

*Each analyst carried out seven complete determinations independently, from weighing through measurement. Values are given to the full precision used in the statistical analysis so that the tests in Table S19 can be reproduced.*

**Table S19.** Intermediate-precision components and analyst-effect tests.

| Element | Intermediate precision RSD (%) | Predicted reproducibility RSD (%) | One-way ANOVA F | One-way ANOVA p | Welch F | Welch $df_2$ | Welch p | $F_{max}$ | Levene p | Bartlett p | Largest analyst-mean difference (% rel.) |
|---|---|---|---|---|---|---|---|---|---|---|---|
| Li | 1.57 | 3.21 | 2.160 | 0.144 | 2.116 | 11.97 | 0.163 | 1.24 | 0.686 | 0.960 | 1.61 |
| Fe | 0.56 | 1.74 | 3.251 | 0.062 | 4.050 | 11.54 | 0.047 | 2.99 | 0.440 | 0.402 | 0.60 |

| Element | Intermediate precision RSD (%) | Predicted reproducibility RSD (%) | One-way ANOVA F | One-way ANOVA p | Welch F | Welch $df_2$ | Welch p | $F_{max}$ | Levene p | Bartlett p | Largest analyst-mean difference (% rel.) |
|---|---|---|---|---|---|---|---|---|---|---|---|
| P | 2.02 | 2.26 | 2.192 | 0.141 | 4.052 | 9.97 | 0.052 | 10.30 | 0.043 | 0.040 | 1.88 |
| Ti | 1.42 | 5.37 | 0.129 | 0.880 | 0.144 | 11.80 | 0.867 | 1.88 | 0.859 | 0.762 | 0.38 |

*Interpretive note for Table S19. Degrees of freedom for the one-way analysis were 2 and 18 (critical F = 3.555). Only P failed the equal-variance checks ($F_{max}$ = 10.30, Levene p = 0.043, Bartlett p = 0.040), so for that element the Welch result is the appropriate test; for Li, Fe and Ti the Welch result is reported as a sensitivity analysis. In every case the largest difference between analyst means remained within the ±2% practical bias interval, and the intermediate-precision RSD remained below the Thompson-modified Horwitz predicted reproducibility.*

## S6. Sensitivity Check and Uncertainty

**Table S20. Ti sensitivity check at approximately 10 mg kg$^{-1}$, measured below the lower calibration limit.**

| No. | Test portion (g) | Measured solution (mg L$^{-1}$) | Calculated Ti (mg kg$^{-1}$) | Reference (mg kg$^{-1}$) |
|---|---|---|---|---|
| 1 | 0.2000 | 0.019 | 9.50 | 10.00 |
| 2 | 0.1992 | 0.019 | 9.54 | 10.04 |
| 3 | 0.1997 | 0.019 | 9.51 | 10.02 |
| 4 | 0.2003 | 0.021 | 10.48 | 9.99 |
| 5 | 0.2004 | 0.019 | 9.48 | 9.98 |
| 6 | 0.1999 | 0.018 | 9.00 | 10.01 |
| 7 | 0.2006 | 0.018 | 8.97 | 9.97 |
| Mean | | | 9.50 | |
| Standard deviation, s | | | 0.499 | |
| RSD (%) | | | 5.25 | |
| Recovery (%) | | | 95.0 | |

*Seven test portions of approximately 0.2 g were spiked at about 10 mg kg$^{-1}$ Ti on a solid basis, digested independently and diluted to 100 mL. The solutions were read against the routine 0.5–5.0 mg L$^{-1}$ calibration, that is, well below the lowest calibration standard and below the level at which the calibration model was shown to fail; in addition, the reported solution concentrations are quantised by the reporting resolution, one increment of 0.001 mg L$^{-1}$ corresponding to 0.5 mg kg$^{-1}$ on a solid basis. No limit of detection or quantification is therefore derived from these data, and the 95.0% recovery is an indicative value obtained outside the calibrated range. The lower calibration limit for Ti is the lowest calibration standard, 0.5 mg L$^{-1}$, equivalent to 250 mg kg$^{-1}$ for this test portion, and results are not reported below it. The Ti content of the commercial sample, approximately 1400 mg kg$^{-1}$, is about 5.6 times that level. Performance at the lower calibration limit itself is reported in Tables S33 and S34.*

**Table S21.** Relative contribution of uncertainty components to the combined variance (%).

| Component | Li | Fe | P | Ti |
|---|---|---|---|---|
| Sample mass | 4.5 | 19.4 | 1.7 | 1.4 |
| Final volume | 0.1 | 0.2 | 0.0 | 3.3 |
| Calibration curve | 22.5 | 47.6 | 87.9 | 84.8 |
| Repeatability | 72.9 | 32.8 | 10.4 | 10.6 |

**Table S22.** Detailed uncertainty budget.

| Quantity | Li | Fe | P | Ti |
|---|---|---|---|---|
| Mean test-portion mass (g) | 0.1015 | 0.1015 | 0.1015 | 0.2007 |
| u(mass) (g) | 0.000144 | 0.000144 | 0.000144 | 0.000175 |

| Quantity | Li | Fe | P | Ti |
|---|---|---|---|---|
| Final volume (mL) | 1000 | 1000 | 1000 | 100 |
| u(volume) (mL) | 0.1479 | 0.1479 | 0.1479 | 0.1351 |
| Calibration concentration (mg $L^{-1}$) | 4.4129 | 33.54 | 19.87 | 2.825 |
| u(calibration) (mg $L^{-1}$) | 0.0140 | 0.0744 | 0.2050 | 0.0194 |
| Repeatability mean (%) | 4.3506 | 33.06 | 19.58 | 0.1407 |
| u(repeatability) (%) | 0.0248 | 0.0608 | 0.0697 | 0.0003 |
| Combined standard uncertainty (%) | 0.0291 | 0.1062 | 0.2156 | 0.00105 |
| Effective degrees of freedom | 10.7 | 28.9 | 24.7 | 26.5 |
| Expanded uncertainty (%, k = 2) | 0.058 | 0.213 | 0.431 | 0.0021 |
| Relative expanded uncertainty (%) | 1.34 | 0.64 | 2.20 | 1.49 |

*Model: w (%) = C · V / ($10^4$ · m). Components common to replicate measurements (mass, volume and calibration) were separated from the random repeatability component to avoid double counting. u(mass) combines the repeatability of replicate weighings ($8.4 \times 10^{-5}$ g, n = 10), the calibration certificate of the check weight ($1.5 \times 10^{-5}$ g), the calibration certificate of the balance ($1.0 \times 10^{-4}$ g) and the balance resolution ($5.8 \times 10^{-5}$ g). u(volume) is not that of a single flask: for the 1000 mL sample dilution it combines the repeatability of the filling operation (0.045 mL, n = 10), the calibration certificate of the flask (±0.18 mL at k = 2, hence 0.090 mL) and a temperature term, while preparation of the calibration standards contributes a further 0.109 mL. Because the flasks are individually calibrated, the certificate value is smaller than the ISO 1042 Class A tolerance would imply. Relative expanded uncertainties are computed from the unrounded combined values. The effective degrees of freedom obtained from the Welch–Satterthwaite relation were 10.7 (Li), 28.9 (Fe), 24.7 (P) and 26.5 (Ti); k = 2 is therefore an approximation to the corresponding Student t values, retained for consistency with routine reporting practice. The budget represents single-analyst, same-day conditions; a routine-conditions estimate is given in Table S31.*

## S7. External Verification

**Table S23.** Comparison of method means and multi-laboratory assigned values.

| Element | Method mean (%) | Assigned value (%) | Assigned U (%, k = 2) | Relative difference (%) | $E_n$ |
|---|---|---|---|---|---|
| Li | 4.3505 | 4.2913 | 0.227 | +1.38 | 0.25 |
| Fe | 33.062 | 33.301 | 0.966 | −0.72 | 0.24 |
| P | 19.582 | 19.590 | 0.909 | −0.04 | 0.01 |
| Ti | 0.14073 | 0.14272 | 0.0034 | −1.39 | 0.49 |

*Property values were assigned through a characterization exercise involving 14–16 accredited laboratories using diverse decomposition and measurement approaches; the assigned values weight laboratories equally regardless of the number of determinations contributed. The component budget of the assigned uncertainties is given in Table S29. Agreement is expressed as the compatibility index $E_n = |D| / \sqrt{(U\_ref^2 + U\_method^2)}$, which folds in the uncertainty of the developed method as well as that of the assigned value. No Grubbs outlier was identified and all laboratory z-scores were within $|z| < 2$; with 14–16 laboratories this criterion is weak evidence of consensus and is reported as a screening step. The three laboratories that applied HF-containing multi-acid digestion returned a mean Ti value of 0.14278%, against 0.14270% for the other eleven. With n = 3 against n = 11 and s = 0.0030%, the smallest difference detectable at 80% power is about 0.0055%, or 3.8% relative, so the comparison excludes a loss on the 5.9% scale observed with aqua regia while leaving residual losses below about 4% unresolved. Recomputing the assigned values with the two author-affiliated laboratories removed changed them by +0.18% (Li), −0.09% (Fe), +0.20% (P) and −0.13% (Ti), and the compatibility indices became 0.22, 0.21, 0.05 and 0.45. All normalised differences are well below unity.*

**Table S24.** Coded laboratory-level results of the interlaboratory characterization exercise used for external verification.

| Lab | Digestion | Technique | n per laboratory | Li (%) | Fe (%) | P (%) | Ti (%) |
|---|---|---|---|---|---|---|---|
| LAB-1 | Closed-vessel microwave | ICP-OES | 3 | 4.323 | 33.533 | 19.485 | 0.13833 |
| LAB-2 | Aqua regia; filter ashed and re-digested in aqua regia | ICP-OES | 3 | 4.271 | 32.503 | 19.860 | 0.14433 |
| LAB-3 | $H_2SO_4$–$HNO_3$–HF–$HClO_4$, taken up in $HNO_3$ | AAS (Li); ICP-OES (Fe, P, Ti) | 2 | 4.405 | 33.900 | 19.800 | 0.14600 |

| Lab | Digestion | Technique | n per laboratory | Li (%) | Fe (%) | P (%) | Ti (%) |
|---|---|---|---|---|---|---|---|
| LAB-4 | Closed-vessel microwave ($HCl–HNO_3–HF$) | ICP-OES | 3 | 4.417 | 33.200 | 19.433 | 0.14233 |
| LAB-5 | Closed-vessel microwave | ICP-OES | 3 | 4.304 | 33.537 | 19.937 | 0.14467 |
| LAB-6 | $H_2SO_4–HF–HCl–HNO_3$ multi-acid | ICP-OES (Li, Ti); volumetric (Fe); gravimetric (P) | 2 | 4.225 | 33.315 | 19.580 | 0.14000 |
| LAB-7 | Closed-vessel microwave | ICP-OES | 1 | 4.480 | 33.800 | 19.600 | 0.14600 |
| LAB-8 | $HNO_3–H_2SO_4$, re-dissolved in aqua regia | ICP-OES | 5 | 4.118 | 32.520 | 19.740 | 0.13860 |
| LAB-9 | Closed-vessel microwave | ICP-OES | 5 | 4.402 | 32.916 | 19.290 | 0.14432 |
| LAB-10 | Closed-vessel microwave | AAS | 3 | 4.167 | 33.100 | – | – |
| LAB-11 | Closed-vessel microwave | ICP-OES | 3 | 4.090 | – | – | – |
| LAB-12 | Closed-vessel microwave | ICP-OES | 5 | 4.080 | 33.680 | 19.220 | 0.14320 |
| LAB-13 | Closed-vessel microwave | ICP-OES | 5 | 4.390 | 33.154 | 19.818 | 0.13820 |
| LAB-14 | Closed-vessel microwave | ICP-OES | 5 | 4.302 | 33.800 | 19.720 | 0.14586 |
| LAB-15 | Closed-vessel microwave | ICP-OES | 5 | 4.397 | 33.331 | 19.487 | 0.14442 |
| LAB-16 | Closed-vessel microwave | ICP-OES | 5 | 4.290 | 33.228 | 19.290 | 0.14176 |
| **Mean** | | | | **4.2913** | **33.3011** | **19.5900** | **0.142716** |
| **SD** | | | | **0.1250** | **0.4296** | **0.2307** | **0.002890** |
| **n** | | | | **16** | **15** | **14** | **14** |

*Laboratory codes are arbitrary and do not indicate the order in which results were received; laboratories are identified only by code in accordance with the terms of their participation. All sixteen laboratories held ISO/IEC 17025 accreditation. Reported values are laboratory means of n independent determinations on the same commercial lot; – indicates that the element was not reported, so the numbers of laboratories contributing to the assigned values were 16 (Li), 15 (Fe), 14 (P) and 14 (Ti). The standard errors of the laboratory-mean distributions (u_char) were 0.0312% (Li), 0.1109% (Fe), 0.0617% (P) and 0.00077% (Ti). No result was rejected by the Grubbs test (maximum G = 1.69, 1.86, 1.60 and 1.56 for Li, Fe, P and Ti against critical values of 2.59, 2.55, 2.51 and 2.51 at $\alpha$ = 0.05), and all laboratory z-scores satisfied $|z| < 2$. The three laboratories whose digestion included hydrofluoric acid (LAB-3, LAB-4 and LAB-6) gave a mean Ti mass fraction of 0.14278% (SD 0.00302%) against 0.14270% (SD 0.00300%) for the remaining eleven, a relative difference of 0.05% (two-sample t = 0.04, p = 0.97). LAB-2 applied aqua regia but ashed the filtered residue and re-digested it before measurement; its result therefore does not correspond to the single-stage open-vessel aqua-regia procedure evaluated in Section 2.1, in which the residue was discarded.*

## S8. Temperature Study, Aqua-Regia Mass Balance and Supplementary Uncertainty Data

### Table S25. Effect of digestion temperature on analyte release (n = 7 per level).

| T (°C) | Li (%) | Fe (%) | P (%) | Ti (%) | Ti RSD (%) | Ti rel. (%) |
|---|---|---|---|---|---|---|
| 220 | 4.3036 ± 0.0516 | 32.6417 ± 0.4178 | 19.4454 ± 0.1808 | 0.12510 ± 0.00973 | 7.78 | 87.7 |
| 230 | 4.2594 ± 0.0656 | 32.7802 ± 0.4688 | 19.5635 ± 0.2152 | 0.12971 ± 0.00428 | 3.30 | 90.9 |
| 235 | 4.3003 ± 0.0357 | 33.1458 ± 0.5635 | 19.5380 ± 0.3673 | 0.13993 ± 0.00388 | 2.77 | 98.1 |
| 240 | 4.3094 ± 0.0375 | 33.2755 ± 0.1630 | 19.5884 ± 0.1822 | 0.14267 ± 0.00107 | 0.75 | 100.0 |
| 245 | 4.3293 ± 0.0368 | 33.2625 ± 0.2894 | 19.4877 ± 0.2066 | 0.14235 ± 0.00066 | 0.46 | 99.8 |

*All digests were filtered under identical conditions irrespective of appearance. Values are mean ± standard deviation. Ti rel. is the Ti mean expressed relative to the 240 °C mean. Welch two-sample tests between adjacent levels gave p = 0.28 (220 vs 230), 0.0005 (230 vs 235), 0.12 (235 vs 240) and 0.52 (240 vs 245).*

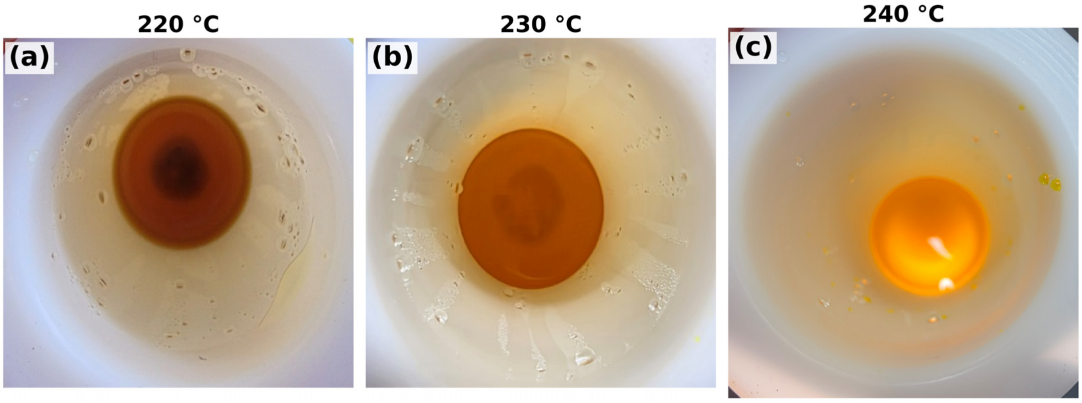


*Figure S1. Appearance of the LFP digests obtained at (a) 220 °C, (b) 230 °C and (c) 240 °C. The quantitative temperature response is shown in Figure 5 of the main text and tabulated in Table S25. Visual clarity alone is not a sufficient endpoint: the 0.29 mg of Ti present in a 0.2 g test portion would not be discernible even if fully retained.*

**Table S26. Aqua-regia filtrate results, individual digestions.**

| Digestion | Test portion (g) | Li (%) | Fe (%) | P (%) | Ti (%) |
|---|---|---|---|---|---|
| 5C-1 | 0.5034 | 4.30302 | 32.13200 | 19.27801 | 0.13097 |
| 5C-2 | 0.4999 | 4.33407 | 32.83482 | 19.31996 | 0.13154 |
| 5C-3 | 0.5000 | 4.30560 | 32.65275 | 19.52070 | 0.13107 |
| 5C-4 | 0.5042 | 4.33633 | 32.77717 | 19.53332 | 0.12978 |
| 5C-5 | 0.5017 | 4.18374 | 31.58984 | 18.95406 | 0.13288 |
| 5C-6 | 0.5000 | 4.32768 | 32.86725 | 19.56720 | 0.12974 |
| 5C-7 | 0.5010 | 4.32088 | 32.66542 | 19.72305 | 0.13420 |
| GF/F-1 | 0.5001 | 4.27163 | 32.08083 | 19.20996 | 0.13444 |
| GF/F-2 | 0.5040 | 4.29698 | 32.49033 | 19.73482 | 0.13066 |
| GF/F-3 | 0.5002 | 4.30664 | 32.59096 | 19.56867 | 0.13456 |
| GF/F-4 | 0.5007 | 4.27845 | 32.51947 | 19.70701 | 0.13251 |
| GF/F-5 | 0.5002 | 4.37469 | 33.12700 | 19.81967 | 0.13495 |
| GF/F-6 | 0.5000 | 4.35804 | 32.81850 | 19.66950 | 0.13417 |
| GF/F-7 | 0.5008 | 4.42364 | 33.44748 | 19.66593 | 0.13233 |
| 5C mean (n = 7) | — | 4.30162 | 32.50275 | 19.41376 | 0.13145 |
| GF/F mean (n = 7) | — | 4.33001 | 32.72494 | 19.62508 | 0.13337 |
| Pooled mean (n = 14) | — | 4.31581 | 32.61384 | 19.51942 | 0.13241 |

*KS L 1629-1 trace-element conditions (0.5 g test portion diluted to 100 mL). Welch tests between the two filter sets gave p = 0.47 (Li), 0.21 (Fe), 0.16 (P) and 0.04 (Ti). The recoveries quoted in the main text are the pooled values.*

**Table S27. Residue mass and residue composition, quantitative cellulose filter set (n = 7).**

| Digestion | Test portion (g) | Residue (mg) | Residue (wt%) | Li (%) | Fe (%) | P (%) | Ti (%) | Ti retained (%) |
|---|---|---|---|---|---|---|---|---|
| AR-1 | 0.5034 | 11.27 | 2.238 | 0.01063 | 0.13965 | 0.13170 | 0.00864 | 6.05 |
| AR-2 | 0.4999 | 9.17 | 1.834 | 0.02375 | 0.23025 | 0.19819 | 0.00920 | 6.45 |
| AR-3 | 0.5000 | 9.27 | 1.853 | 0.01630 | 0.17465 | 0.15125 | 0.00815 | 5.71 |
| AR-4 | 0.5042 | 9.77 | 1.937 | 0.02271 | 0.22417 | 0.19114 | 0.00863 | 6.05 |
| AR-5 | 0.5017 | 10.37 | 2.066 | 0.01540 | 0.16245 | 0.15423 | 0.00752 | 5.27 |
| AR-6 | 0.5000 | 10.77 | 2.153 | 0.01580 | 0.16705 | 0.15400 | 0.00810 | 5.68 |
| AR-7 | 0.5010 | 11.37 | 2.269 | 0.01163 | 0.13762 | 0.13418 | 0.00828 | 5.80 |
| Mean | — | — | 2.050 | 0.01660 | 0.17655 | 0.15924 | 0.00836 | 5.86 |
| SD | — | — | 0.179 | 0.00502 | 0.03724 | 0.02594 | 0.00053 | 0.37 |

*Residue mass corrected for the mean blank-filter change of 1.03 mg (n = 3). Analyte columns are residue contents expressed on the basis of the original test portion. Filtrate and residue were obtained from the same test portion, so the mass balance can be closed sample by sample.*

**Table S28. Residue mass and loss on ignition, glass-fibre filter set (n = 7).**

| Digestion | Test portion (g) | $w_0$ (g) | $w_1$ (g) | $w_2$ (g) | Residue (mg) | Residue (wt%) | LOI (mg) | LOI (wt% of sample) |
|---|---|---|---|---|---|---|---|---|
| GF-1 | 0.5001 | 0.1346 | 0.1449 | 0.1356 | 10.17 | 2.033 | 8.67 | 1.733 |
| GF-2 | 0.5040 | 0.1387 | 0.1498 | 0.1401 | 10.97 | 2.176 | 9.07 | 1.799 |
| GF-3 | 0.5002 | 0.1358 | 0.1471 | 0.1372 | 11.17 | 2.232 | 9.27 | 1.853 |
| GF-4 | 0.5007 | 0.1356 | 0.1466 | 0.1373 | 10.87 | 2.170 | 8.67 | 1.731 |
| GF-5 | 0.5002 | 0.1365 | 0.1483 | 0.1378 | 11.67 | 2.332 | 9.87 | 1.973 |
| GF-6 | 0.5000 | 0.1369 | 0.1495 | 0.1388 | 12.47 | 2.493 | 10.07 | 2.013 |
| GF-7 | 0.5008 | 0.1357 | 0.1464 | 0.1368 | 10.57 | 2.110 | 8.97 | 1.790 |
| Mean | — | — | — | — | — | 2.221 | — | 1.842 |
| SD | — | — | — | — | — | 0.152 | — | 0.112 |

*$w_0$ after ignition at 500 °C for 1 h; $w_1$ after drying at 105 °C to constant mass; $w_2$ after ignition at 500 °C for 2 h. Blank corrections: 0.13 mg for residue mass and 0.63 mg for loss on ignition (n = 3). Loss on ignition includes adsorbed water and the decomposition of residual nitrate and is not equated with carbon content. Taking the assigned total carbon content of 1.4846% as an upper bound, carbon can account for at most 72.4 wt% of the residue, which is compatible with the 73.03 ± 2.65 wt% returned by EDS in Table S1. Applying the localized EDS compositions to the bulk residue mass does not, however, quantitatively reproduce the ICP-OES mass balance for the metals; see Section 2.1 of the main text.*

### Table S29. Uncertainty budget of the assigned property values.

| Component | Assigned value (%) | u_char (%) | u_bb (%) | u_lts (%) | u_sts (%) | U (%, k = 2) |
|---|---|---|---|---|---|---|
| Li | 4.2913 | 0.0312 | 0.0107 | 0.107 | 0.0188 | 0.227 |
| Fe | 33.3011 | 0.1109 | 0.1589 | 0.431 | 0.0989 | 0.966 |
| P | 19.5900 | 0.0617 | 0.1881 | 0.405 | 0.0588 | 0.909 |
| Ti | 0.142716 | 0.00077 | 0.00046 | 0.0014 | 0.00041 | 0.0034 |
| C | 1.4846 | 0.01508 | 0.00194 | 0.0057 | 0.0012 | 0.0326 |

*Between-bottle homogeneity was evaluated on ten bottles with three determinations each; long-term stability over six months at 20 °C by linear regression of the observed trend; and short-term stability at −10, 20 and 50 °C over two weeks, covering transport and short-term storage. u_sts was evaluated from the slope of mass fraction against storage time, as the standard error of that slope multiplied by a two-week exposure, and the largest value across the three storage conditions was adopted; no slope differed significantly from zero (p = 0.42–0.98), but the component is carried in the budget rather than set to zero on that basis. Relative contributions to the combined variance are, for Li, Fe, P, Ti and C respectively: u_char 7.6, 5.3, 1.8, 20.2 and 85.8%; u_bb 0.9, 10.8, 17.1, 7.2 and 1.4%; u_lts 88.8, 79.7, 79.4, 66.8 and 12.3%; u_sts 2.7, 4.2, 1.7, 5.8 and 0.5%. Long-term stability dominates for the four analytes; its magnitude reflects the classical design of the stability study, in which successive time points were measured in different analytical runs, so that run-to-run variation is carried into the standard error of the fitted slope. The underlying homogeneity and stability datasets of this candidate reference material are available from the corresponding author on reasonable request.*

### Table S30. Linking of the two measurement sessions.

| Element | Temperature-study session, 240 °C (n = 7) | Aqua-regia session, re-measured (n = 3) | Difference (%) | p |
|---|---|---|---|---|
| Li | 4.30943 ± 0.03769 | 4.30891 ± 0.02026 | -0.01 | 0.98 |
| Fe | 33.27549 ± 0.16439 | 33.29233 ± 0.02658 | +0.05 | 0.80 |
| P | 19.58842 ± 0.18173 | 19.52117 ± 0.14703 | -0.34 | 0.57 |
| Ti | 0.14267 ± 0.00107 | 0.14331 ± 0.00224 | +0.45 | 0.67 |

*Three digests from the 240 °C level of the temperature study were re-measured within the aqua-regia session against that session's calibration. For each element, the absolute relative difference was smaller than the corresponding intermediate-precision RSD reported in Table S19, and none of the differences was statistically significant.*

### Table S31. Uncertainty under routine conditions.

| Element | Intermediate-precision RSD (%) | Observed bias (%) | u_routine (%) | U_routine (%, k = 2) | U_single-day (%, k = 2) |
|---|---|---|---|---|---|
| Li | 1.57 | −0.2 | 1.58 | 3.17 | 1.34 |
| Fe | 0.56 | −0.7 | 0.90 | 1.79 | 0.64 |
| P | 2.02 | −1.0 | 2.25 | 4.51 | 2.20 |
| Ti | 1.42 | −0.9 | 1.68 | 3.36 | 1.49 |

*Derived following ISO 21748:2017 and the Eurachem/CITAC guide by combining the intermediate-precision variance with an uncorrected-bias term, and reported alongside the single-day GUM budget of Table S22.*

## S9. Instrumental Conditions, Lower-Limit Performance, Specification Limits and Material Characterization

### Table S32. ICP-OES operating conditions.

| Parameter | Condition |
|---|---|
| Instrument | Avio 500 (PerkinElmer, USA) |
| RF generator | 40 MHz |
| RF power | 1500 W |
| Plasma gas flow | 12 L min$^{-1}$ |
| Auxiliary gas flow | 0.2 L min$^{-1}$ |
| Nebulizer gas flow | 0.55 L min$^{-1}$ |
| Nebulizer | Miramist |
| Viewing mode | Radial (Li, Fe, Ti); axial (P 178.221 nm) |
| Sample uptake | 1.5 mL min$^{-1}$ |
| Internal standard | Y, 5 mg L$^{-1}$ (371.029 nm) |
| Analytical lines | Li 670.784; Fe 259.939; P 178.221; Ti 334.940 nm |
| Optical purge gas | Argon; supply pressure 0.4–0.5 MPa; normal flow setting |
| Shear gas | Argon |
| Background correction | 2-point, all lines |
| Interelement correction | Not applied |
| Peak algorithm | Peak area, 3 points per peak |
| Read time / delay | Auto 1–5 s / 60 s |
| Replicates per solution | 3 |

*The measurement procedure is described in Section 3.4 of the main text.*

### Table S33. Verification at the lower calibration limit for Ti: matrix-matched check solution at 0.5 mg L$^{-1}$ (n = 7).

| Replicate | Found (mg L$^{-1}$) | Solid-basis equivalent (mg kg$^{-1}$) | Recovery (%) |
|---|---|---|---|
| 1 | 0.495 | 247.5 | 99.0 |
| 2 | 0.504 | 252.0 | 100.8 |
| 3 | 0.507 | 253.5 | 101.4 |
| 4 | 0.493 | 246.5 | 98.6 |
| 5 | 0.508 | 254.0 | 101.6 |
| 6 | 0.493 | 246.5 | 98.6 |
| 7 | 0.497 | 248.5 | 99.4 |
| Mean | 0.4996 | 249.8 | 99.9 |
| SD | 0.0066 | 3.3 | – |
| RSD (%) | 1.32 | 1.32 | – |
| Acceptance criterion | – | – | Recovery 95–105%; RSD ≤ 10% |

*The check solution was prepared from a stock solution independent of that used for the calibration standards, matched to the acid and matrix composition of the Ti sample solutions, and measured against the routine 0.5–5.0 mg L$^{-1}$ calibration. The solid-basis equivalent corresponds to a 0.2 g test portion diluted to 100 mL, so 0.5 mg L$^{-1}$ corresponds to the lower calibration limit of 250 mg kg$^{-1}$. Both criteria were met.*

**Table S34. Relative standard uncertainty of a single Ti result at the lower calibration limit and at the Ti content of the investigated material.**

| Component | At 250 mg kg$^{-1}$ (0.5 mg L$^{-1}$) (%) | At ≈1410 mg kg$^{-1}$ (2.82 mg L$^{-1}$) (%) |
|---|---|---|
| Test-portion mass | 0.088 | 0.087 |
| Final volume | 0.135 | 0.135 |
| Calibration prediction | 4.09 | 0.69 |
| Repeatability, single result | 1.46 | 0.64 |
| Combined standard uncertainty | 4.34 | 0.95 |
| Expanded uncertainty, k = 2 | 8.7 | 1.9 |
| Expanded uncertainty, k = 2 (mg kg$^{-1}$) | 22 | 27 |
| Share of variance from calibration prediction | 88% | 52% |

*Same measurement model, mass and volume terms and calibration-prediction formula as Table S22, evaluated at the indicated solution concentrations. The values refer to a single result rather than to the mean of seven, so repeatability enters as a standard deviation rather than as a standard error. At the lower calibration limit the repeatability combines the RSD of the check solution in Table S33 (1.32%) with the complete-method repeatability RSD at the sample level (0.64%, Table S14) as a conservative allowance for the digestion step; at the sample level the complete-method repeatability RSD is used. Because the lower-limit check was made on a solution, the value at 250 mg kg$^{-1}$ is a model-based estimate for the investigated matrix. Calibration prediction dominates at the lower limit because the prediction interval widens toward the end of the calibration range.*

**Table S35. Illustrative specification half-widths under the authors' criterion of a half-width of at least four times the expanded uncertainty.**

| Element | U, routine (%, k = 2) | Minimum half-width (% rel.) | Half-width at assigned value (%, absolute) |
|---|---|---|---|
| Li | 3.17 | ±12.7 | ±0.54 |
| Fe | 1.79 | ±7.2 | ±2.38 |
| P | 4.51 | ±18.0 | ±3.53 |
| Ti | 3.36 | ±13.4 | ±0.019 |

*Routine-condition expanded uncertainties from Table S31, which apply to a single result. With a guard band equal to the expanded uncertainty (Eurachem/CITAC Guide: Use of Uncertainty Information in Compliance Assessment, 2nd ed., 2021), a half-width of at least four times the expanded uncertainty, a conservative criterion adopted by the authors, keeps the guard band within one quarter of the specification half-width. No public product specification exists for this material; the values indicate the narrowest specification against which the method can make release decisions without the guard band dominating the acceptance interval, and should be compared with the specification of the user's own product.*

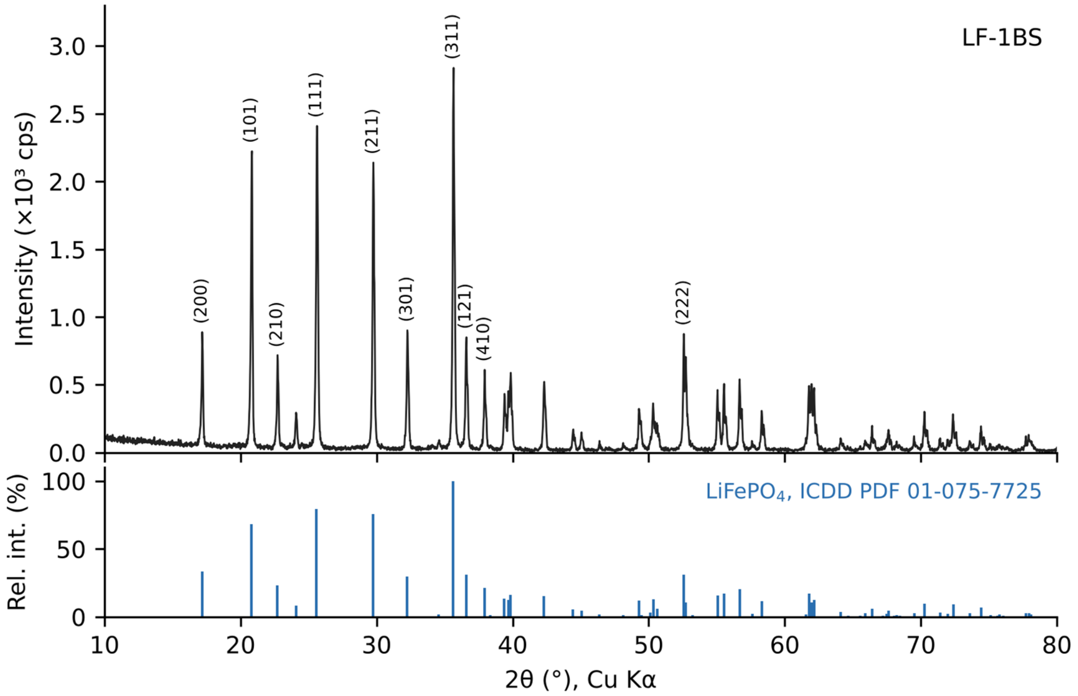


*Figure S2. Powder X-ray diffraction pattern of the investigated Ti-doped LFP (LF-1BS; upper panel) and the reference pattern of olivine $LiFePO_4$ (triphylite, orthorhombic, Pnma; ICDD PDF 01-075-7725; lower panel). All reflections are indexed to $LiFePO_4$, including the Cu $K\alpha_2$ components at higher angles, and no reflection of a crystalline impurity phase is observed. The Halder–Wagner method gave an apparent crystallite size of about 64 nm and a microstrain of about 0.05%. Selected reflections are labelled with their hkl indices.*

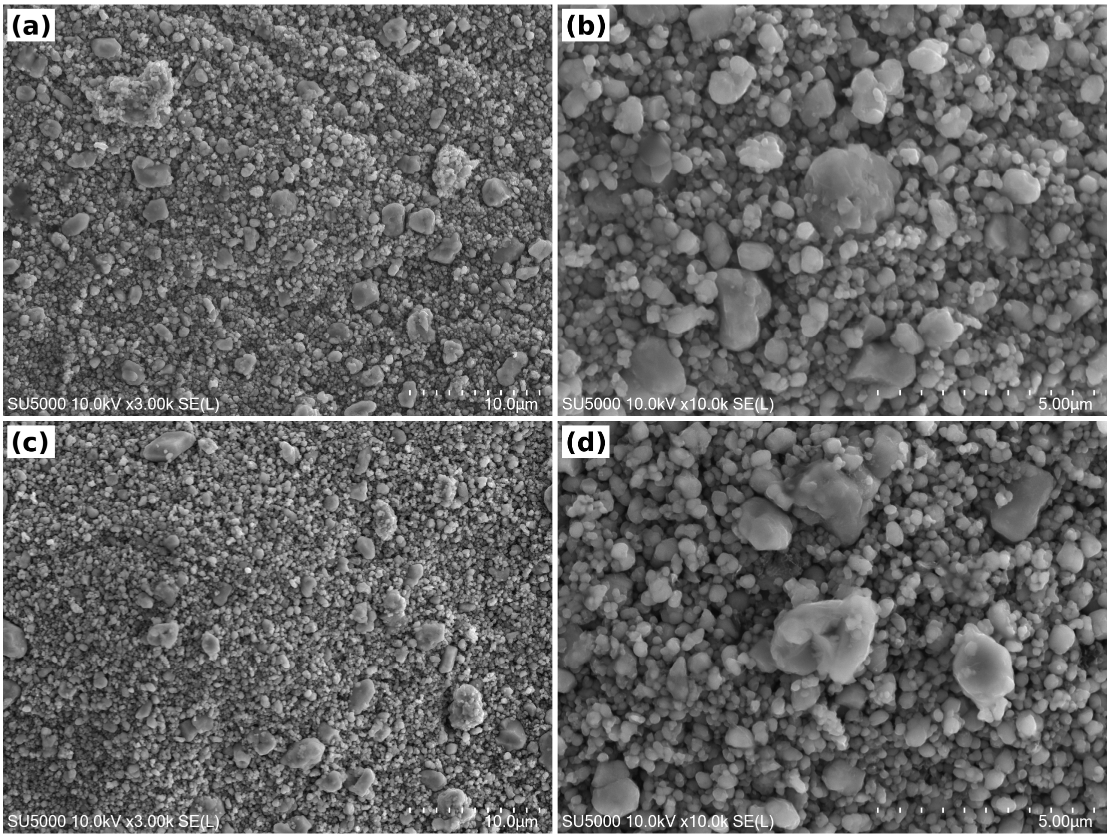


*Figure S3. FE-SEM secondary-electron images of the investigated Ti-doped LFP (LF-1BS) in two areas at (a,c) ×3000 and (b,d) ×10,000 (Hitachi SU5000, 10 kV, uncoated powder on carbon tape). The powder consists of rounded primary particles, mostly 0.2–0.5 µm across, with a smaller number of dense particles of 1–2 µm and loosely bound clusters of fine particles.*

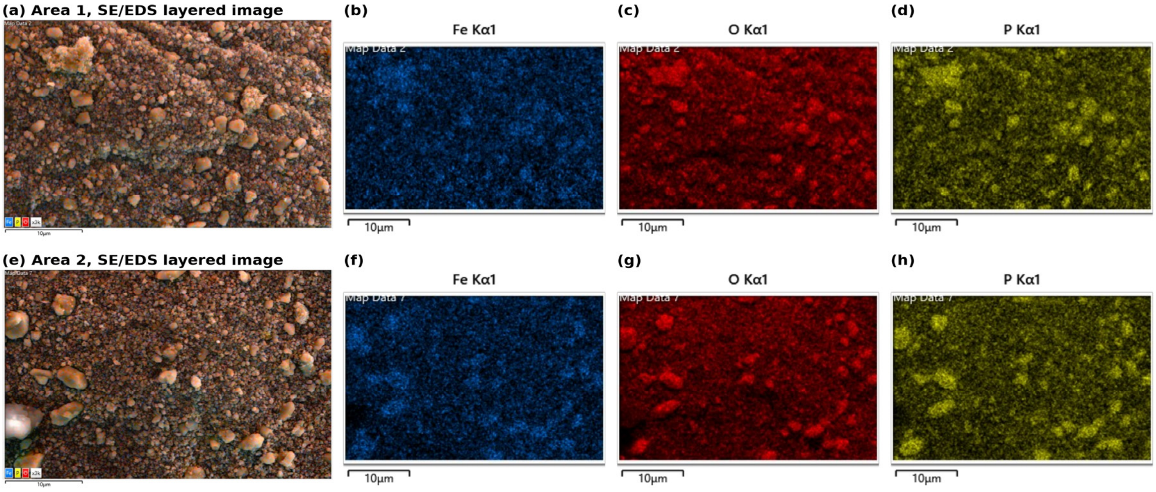


*Figure S4. SE/EDS layered images (a,e) and Fe Kα (b,f), O Kα (c,g) and P Kα (d,h) maps of two areas of the investigated material (×3000, 15 kV). Fe, P and O are co-distributed over the particles; local intensity variations follow particle topography. Li could not be mapped because its X-ray energy lies below the low-energy limit of the EDS detector used, and Ti was not identified in the map-sum spectra, consistent with its content (about 0.14%) lying at or below the EDS detection limit. C was excluded because the powder was mounted on carbon tape. Map-sum compositions are given in Table S36; homogeneity at the scale of the analytical test portion is established by the between-bottle study (Table S29).*

**Table S36. EDS map-sum composition of the investigated material (mass fraction, %, normalized to O + P + Fe).**

| Map | O | P | Fe | Fe/P (atomic) |
|---|---|---|---|---|
| Area 1, ×3000 | 47.30 | 18.26 | 34.44 | 1.05 |
| Area 1, ×5000 | 46.28 | 18.72 | 34.99 | 1.04 |
| Area 2, ×3000 | 46.35 | 18.65 | 35.00 | 1.04 |
| Area 2, ×5000 | 46.54 | 18.37 | 35.09 | 1.06 |
| Mean | 46.62 | 18.50 | 34.88 | 1.05 |
| RSD (%) | 1.0 | 1.2 | 0.85 | 1.0 |

*Hitachi SU5000 with an Ultim Max silicon drift EDS detector (Oxford Instruments), 15 kV, working distance 20 mm, uncoated powder on carbon tape. C was excluded from the quantification because of the substrate, Li is not detectable with the detector used, and Ti was not identified in the spectra. EDS is semi-quantitative and normalized to 100%; the ×5000 maps are not shown in Figure S4; the values are used to compare areas and magnifications, not to determine the composition, which is given by the ICP-OES results in the main text.*
